# Genome skimming of Poecilosclerida (Porifera) reveals conserved and hyperrearranged mitochondrial genomes

**DOI:** 10.7717/peerj.21781

**Published:** 2026-11-11

**Authors:** Thiago Silva de Paula, Dora de Moura Barbosa Leite, Fabiano Thompson, Eduardo Hajdu

**Affiliations:** 1 Departamento de Genética, Universidade do Estado do Rio de Janeiro, Rio de Janeiro, Brazil; 2 Museu Nacional, Universidade Federal do Rio de Janeiro, Rio de Janeiro, Brazil; 3 Departamento de Biologia Marinha, Universidade Federal do Rio de Janeiro, Rio de Janeiro, Brazil

**Keywords:** Marine invertebrates, Demospongiae, Comparative organellar genomics, Gene order, Gene rearrangement, Gene fusion

## Abstract

Genome skimming is a cost-effective approach that efficiently recovers multi-copy genomic regions, making it particularly valuable for phylogenetic research and biodiversity assessments. In this study, we applied shallow shotgun metagenome sequencing to 15 poecilosclerid sponges, successfully assembling complete mitochondrial genomes for four species and full rDNA clusters for the majority of samples, despite considerable variation in the retrieval of genomic regions and exogenous, non-poriferan sequences. Phylogenetic reconstructions using both rDNA and mtDNA sequences uncovered extensive polyphyly within several key families, such as Coelosphaeridae and Mycalidae, underscoring significant systematic inconsistencies at generic, familial, and ordinal levels, and emphasizing the need for comprehensive taxonomic revision throughout Poecilosclerida to reflect natural groups. The mitogenomes obtained from *Clathria* (*Microciona*) sp. (18,725 bp), *Desmapsamma anchorata* (19,099 bp), *Latrunculia biformis* (19,630 bp), and *Neopodospongia tupecomareni* (19,983 bp) exhibited conserved architectural features typical of the order, alongside notable variations such as gene overlaps, non-canonical start codons, and an unusual fused *nd3*::*nd4L* gene. Our findings highlight the applicability of genome skimming for phylogenetics and metagenomic insights, though success can vary due to biological and methodological factors. This approach has proven to be a cost-effective and powerful tool for advancing sponge systematics, despite challenges in assembling hyperrearranged mitogenomes in certain lineages, paving the way for broader genomic surveys in non-model organisms.

## Introduction

Genome skimming has been proved a cost-effective and efficient approach for evolutionary and systematic studies (Dodsworth, 2015; Trevisan et al., 2019). Although firstly described for plants (Straub et al., 2012), genome skimming has become a widely popular method across fields and organisms (*e.g.*, Richter et al., 2015; Denver et al., 2016; Lu et al., 2025; Turner et al., 2025), with dozens of studies published every year. By generating sequences from whole genomes at low coverage, this method has the potential to recover highly represented fractions of the genomes, such as multi-copy regions. Thus, researchers can easily recover full ribosomal (rDNA), mitochondrial (mtDNA), and chloroplast (cpDNA) DNA sequences for conducting phylogenetic analyses, even from museum preserved specimens (Ripma, Simpson & Hasenstab-Lehman, 2014), biodiversity assessments (Gastineau et al., 2025), and comparative genomics (Linard et al., 2015; Denver et al., 2016; Lu et al., 2022). The method is particularly useful for non-model organisms, especially due to its affordability and scalability, making it a powerful tool for large-scale systematic studies and biodiversity surveys (*e.g.*, Nevill et al., 2020; Hoban et al., 2022).

Sponges (Porifera) are known for their unusual mitochondrial genome structures (Lavrov & Pett, 2016; Lavrov et al., 2023), rich and complex metabolite compositions (Berne et al., 2016; Zhang et al., 2022), and intricate symbiotic associations with diverse microbial communities (Webster & Thomas, 2016; Nascimento-Silva, Hardoim & Custódio, 2022). For these reasons, sponge studies using targeted or enrichment methods, such as LA-PCR, are usually species-specific and often fail to generate results (Lavrov et al., 2023; Ahmed, Kayal & Lavrov, 2024). By allowing us to circumvent these intrinsic difficulties from sponge biology, at a feasible cost, genome skimming can be a powerful (and promising) tool for studying sponges. Regarding the holobiont nature of sponges, a genome skim from these animals would also contain sequences from highly abundant microorganisms in association. In providing a sample of the sponge metagenome (*viz.*, shallow shotgun metagenome sequencing, SSMS), the method can aid in (i) deciphering host-symbiont interactions (Robbins et al., 2021), (ii) identifying biosynthetic gene clusters linked to symbiont-derived bioactive compounds (Loureiro et al., 2022), and (iii) the offer of insights into the sponge ecological and biotechnological potential (García-Bonilla et al., 2021), notably in cases when symbionts cannot be cultured (Dat et al., 2021). Thus, genome skimming provides a cost-effective strategy to explore sponge biodiversity, evolutionary history, and symbiotic networks without the need for the costly whole-genome sequencing or the frustrating targeted methods (*e.g.*, Turner et al., 2025).

In the last decade, phylogenetic studies using complete rDNA and mtDNA sequences have underscored how molecular data is useful to solve deep phylogenetic relationships among sponge taxa (*e.g.*, Redmond et al., 2013; Thacker et al., 2013; Lavrov et al., 2023), making genome skimming highly appealing. Evolutionary studies on sponges are often complicated by the loss of phylogenetic signal from the distant time or the heterogeneity in evolutionary rates across groups (Wörheide et al., 2012; Feuda et al., 2017; Simion et al., 2017). Combined with homoplasy in traditional morphological traits (*e.g.*, spicules), they have driven redefinitions for several demosponge clades (Morrow & Cárdenas, 2015). The subordinal classification of Poecilosclerida provides a notable example of incongruency between molecular and morphological data (Hajdu et al., 2013), in which current classification schemes persist in part because not enough representatives have been incorporated into a phylogenetic framework. Poecilosclerida is currently the richest and most diverse clade of sponges (De Voogd et al., 2025), with over 2,500 species, surpassing even classes of calcareous (838 spp.) and glass (715 spp.) sponges. Thus, sampling as much of this diversity and integrating it into phylogenetic analyses is crucial to reliably reconcile morphological and molecular evidence for a revised classification of higher taxa within the Poecilosclerida.

In this study we performed genome skimming (SSMS) of fifteen poecilosclerid sponges, spanning ten Poecilosclerida families and one Merliida family. We attempted to retrieve complete rDNA and mtDNA from sequence reads in order to complement currently available data for phylogenetic analyses. The organization of these genomic regions and the classification of Poecilosclerida were discussed in detail, and despite some flaws in the approach, we concluded that genome skimming can be instrumental in resolving problematic evolutionary relationships in Porifera.

## Methods

### Sampling, DNA extraction, library preparation and shallow shotgun sequencing

Fifteen samples of poecilosclerid sponges were obtained through different collection methods over the years across several expeditions of the Laboratório de Taxonomia Integrativa de Porifera (TAXPO, MN/UFRJ) and through tissue donations from collaborators from other institutions (Table 1; Fig. 1). Whenever possible, samples were immediately preserved in RNAlater^®^ (Thermo Fisher Scientific, Waltham, MA, USA), and kept refrigerated at −20 °C in the laboratory. Species identification was carried out during the deposit of voucher specimens by the collection curators, using traditional morphological characteristics, including examination of histological slides of spicule dissociation and skeletal sections. Total genomic DNA purifications were conducted at the Laboratório de Genética Marinha (UERJ), following the same methodology as in De Paula et al. (2024) and DNA concentrations were estimated with Qubit (Thermo Fisher Scientific) at Laboratório de Microbiologia (UFRJ). Samples were sent to the Rush University Genomics and Microbiome Core Facility (GMCF, Chicago, IL, USA) for library preparation with 100 ng of DNA and the Illumina DNA Prep kit (Illumina, Inc., San Diego, CA, USA; previously known as Nextera DNA Flex Library Prep). Shallow shotgun metagenomic sequencing (SSMS) was conducted with 2 × 150 bp paired-end sequencing on an Illumina NovaSeq 6000 system (Illumina, Inc., San Diego, CA, USA) according to the workflow indicated by the manufacturer, aiming to obtain 1 Gb of data per sample, or roughly 6% of a SP flow cell for all samples.

**Table 1:** Poecilosclerida sponge samples used for genome skimming.

**Species**	**Code**	**Voucher**	**Locality**	**Depth**	**Coordinates**	**Identifier**	**Date**	**DNA conc.**
*Asbestopluma pennatula* (Schmidt, 1875)	Ape	ZMBN 148461	Norway: Vesterålen Archipel., Andøya Isl., Bleik Canyon	1˜000 m	69.4953 N, 15.6494 E	H.T. Rapp & J. Hestetun	2018-07-30	>600.0 ng/μ L
*Chondrocladia grandis* (Verrill, 1879)	Cgr	ZMBN 148469	Norway: Vesterålen Archipel., Andøya Isl., Bleik Canyon	1˜000 m	69.4953 N, 15.6494 E	H.T. Rapp & J. Hestetun	2018-07-30	118.0 ng/μ L
*Cladorhiza gelida* Lundbeck, 1905	Cge	ZMBN 148466	Norway: Vesterålen Archipel., Andøya Isl., Bleik Canyon	1˜000 m	69.4953 N, 15.6494 E	H.T. Rapp & J. Hestetun	2018-07-30	118.0 ng/μ L
*Clathria* (*Microciona*) sp.	CMsp	MNRJ 20519	Brazil: Bahia, Porto Seguro, Arraial D’Ajuda	0 m	15.502317 S, 38.9322 W	D. Leite & E. Hajdu	2019-03-20	84.4 ng/μ L
*Desmapsamma anchorata* (Carter, 1882)	Dan	MNRJ 22097	Brazil: Rio de Janeiro, Angra dos Reis, Saco da Ribeira	6.6 m	23.021236 S, 44.329900 W	E. Hajdu & D. Leite	2019-01-12	20.8 ng/μ L
*Diacarnus megaspinorhabdosa* Kelly-Borges & Vacelet, 1995	Dme	ZMAPOR 13278	Indonesia: Celebes	9 m	–	N.J. de Voogd	1997-04-07	47.4 ng/μ L
*Guitarra sepia* Lerner et al., 2004	Gse	MNRJ 22099	Brazil: Rio de Janeiro, Cabo Frio	7.5 m	–	E. Hajdu	2019-01-24	>600.0 ng/μ L
*Hamacantha falcula* (Bowerbank, 1874)	Hfa	ZMAPOR 19419	Ireland	587 m	55.499 N, 15.798 W	R.W.M. van Soest	2005-06-25	72.8 ng/μ L
*Latrunculia biformis* Kirkpatrick, 1908	Lbi	MNRJ 11166	Antarctic: South Shetlands Archipel., King George Isl.	300 m	62.140145 S, 58.435199 W	E. Hajdu	2009-12-11	48.2 ng/μ L
*Lissodendoryx isodictyalis* (Carter, 1882)	Lis	MNRJ 10474	Brazil: Bahia, Salvador, Ponta do Humaitá	1.5 m	12.928032 S, 38.519347 W	E. Hajdu	2006-03-12	62.8 ng/μ L
*Mycale* (*Grapelia*) sp.	MGsp	MNRJ 22646	Panama: Bocas del Toro Pastores Isl.	2 m	9.23981 N, 82.33416 W	E. Hajdu & D. Leite	2019-07-13	>600.0 ng/μ L
*Mycale* (*Naviculina*) *diversisigmata* (van Soest, 1984)	Mdi	MNRJ 22115	Brazil: Alagoas, Maceió, Ponta Prego	0 m	9.530211 S, 35.591511 W	E. Hajdu	2019-02-22	78.6 ng/μ L
*Neopodospongia tupecomareni* Hajdu et al., 2013	Ntu	MNRJ 10323b	Chile: Quintupeu Fjord	25 m	42.164956 S, 72.430319 W	E. Hajdu & Ph. Willenz	2006-10-10	56.0 ng/μ L
*Phlyctaenopora* (*Barbozia*) sp.	PBsp	MNRJ 17428 (= WAM Z 35503)	Australia: Western Australia	85–92 m	29.8028 S, 114.4389 E	J. Fromont & C.S. Whisson	2005-12-02	>600.0 ng/μ L
*Ulosa digitata* (Esper, 1794)	Udi	ZMAPOR 14109^*^	France: Bretagne	25 m	48.688 N, 3.884 W	R.W.M. van Soest	1998-09-11	57.2 ng/μ L

**Notes.**

ZMBN Zoological Museum in Bergen, Norway MNRJ Museu Nacional, Universidade Federal do Rio de Janeiro, Brazil ZMAPOR Zoölogisch Museum van de Universiteit van Amsterdam, Netherlands

* Originally identified as *Ulosa stuposa* (Esper, 1794).

**Figure 1:**
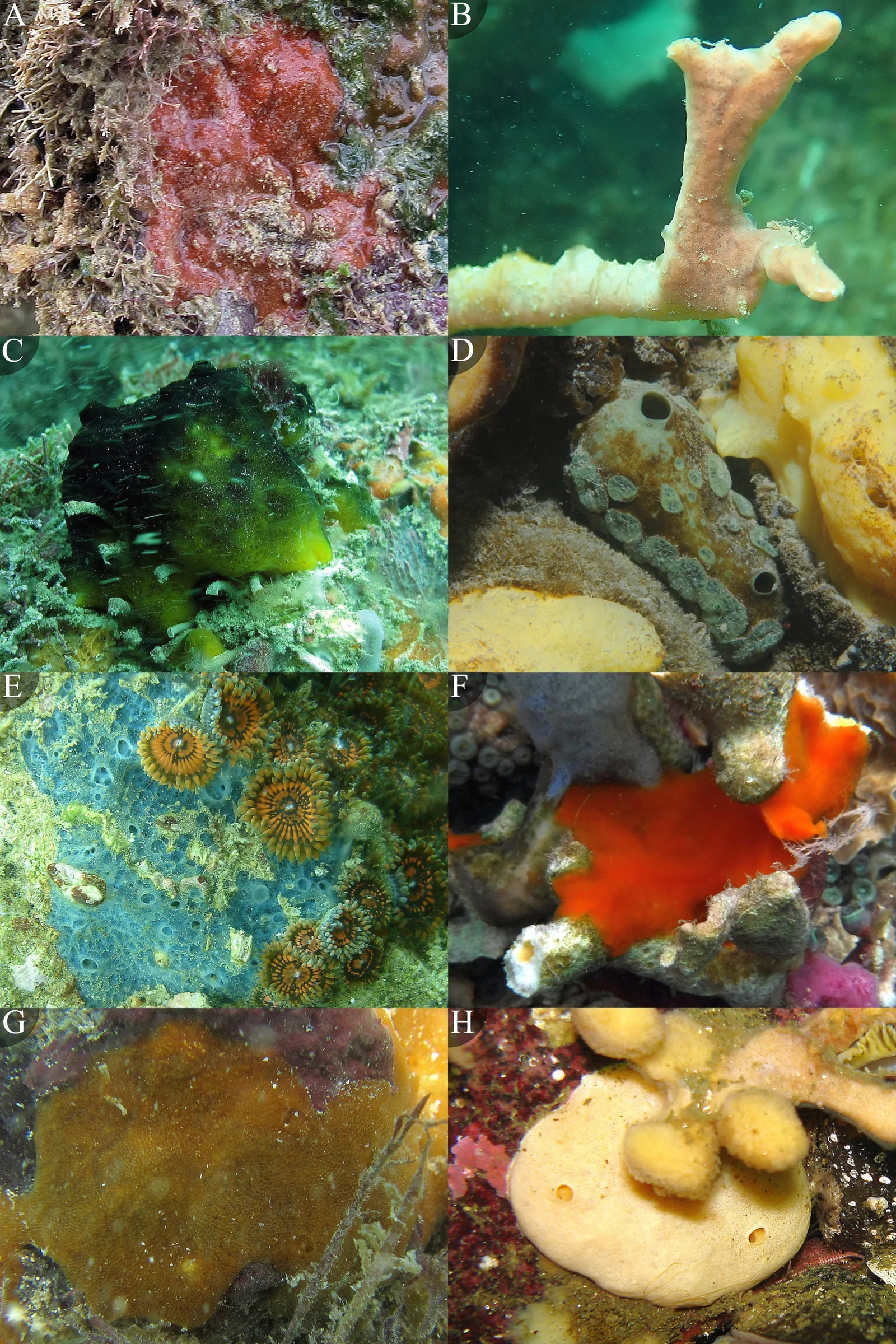
Live images of part of the studied species. (A) *Clathria* (*Microciona*) sp., w/ cleistochelae, MNRJ 20519. (B) *Desmapsamma anchorata* (Carter, 1882), MNRJ 22097. (C) *Guitarra sepia* Lerner, Hajdu, Custódio & van Soest, 2004, MNRJ 11210*. (D) *Latrunculia* (*Aciculatrunculia*) *biformis* Kirkpatrick, 1908, MNRJ 13817*. (E) *Lissodendoryx isodictyalis* (Carter, 1882), MNRJ 10474. (F) *Mycale* (*Grapelia*) sp., MNRJ 22646. (G) *Mycale* (*Naviculina*) *diversisigmata* (van Soest, 1984), MNRJ 22115. (H) *Neopodospongia tupecomareni* Hajdu, Desqueyroux-Faúndez, Carvalho, Lôbo-Hajdu & Willenz, 2013, MNRJ 10323b. Representative specimens (*) from the same species and locality. Specimens lacking underwater photographs are not illustrated here.

### Quality control, mapping, assembly, annotation and analysis

Quality control, mapping, assembly, and annotation were performed as previously described in De Paula et al. (2024). Briefly, reads were filtered with Trimmomatic v0.39 (Bolger, Lohse & Usadel, 2014), additionally trimmed with the ‘bbduk’ script from BBTools package v37.62 (Brian Bushnell, available at https://sourceforge.net/projects/bbmap/), and mapped against rDNA and mtDNA references with the ‘bbmap’ script. Mapped reads were de novo assembled with Megahit v1.2.9 (Li et al., 2015). Mitochondrial DNA sequences were annotated for protein-coding genes (PCGs) with ORF Finder (https://www.ncbi.nlm.nih.gov/orffinder/), for tRNAs with tRNAscan-SE v2.0 (Chan et al., 2021), and for rRNAs by BLAST and multiple sequence alignments (MSA) with MAFFT v7.505 (Katoh, Rozewicki & Yamada, 2019). Final annotations were verified with MITOS2 (Donath et al., 2019). Composition skew values were computed according to Lobry (1996) and nucleotide and amino acid compositions and codon usage were obtained with SMS v2 (Stothard, 2000). Gene maps were generated with CGView v2.0.3 (Stothard & Wishart, 2005). When initial assemblies failed to recover complete mtDNA sequences, the resulting contigs were used as reference sequences in iterative rounds of mapping and assembly. When retrieving nearly full sequences, those were aligned against *Negombata magnifica* mtDNA (NC_010171) in order to estimate missing nucleotides and circularization. When this approach failed to improve the assembly further, filtered reads were mapped against individual mtDNA genes for assembly of sequences to be used in phylogenetic analyses. The same methods were used to recover mitochondrial gene sequences from previously published, unannotated Poecilosclerida mitogenomes in databases (Table S1). Additionally, we also recovered the nuclear 45S rDNA cluster from available transcriptomic data for phylogenetic analysis (Table S2). Low complexity reads were estimated using ‘bbduk’ script from the BBTools package v37.62 (Brian Bushnell, available at https://sourceforge.net/projects/bbmap/) with entropy level set to 0.7. The software Kraken2 (Wood & Salzberg, 2014) was used to classify reads from non-Poriferan sources, *viz.*, bacteria, archaea, viruses, plasmids, human, protists, and fungi (PlusPF database v.2022-06-07, confidence 0.1) implemented within the Galaxy framework (The Galaxy Community, 2024). To estimate the proportion of reads from the sponge host, reads were mapped against the concatenated genomes of *Crambe crambe* (GCF_963924555), *Diacarnus erythraeanus* (GCF_964016965), and *Lycopodina hypogea* (GCF_963969325). Multivariate analyses were performed in R using the ‘vegan’ package (Oksanen et al., 2025), with Detrended Correspondence Analysis (DCA) applied to metagenome composition derived from Kraken2 results (above) and Principal Component Analysis (PCA) used to examine amino acid composition patterns across Poecilosclerid mitogenomes (see below). RNA secondary structures were drawn with the RNAfold WebServer (Gruber, Bernhart & Lorenz, 2015). The similarity plot was drawn with SimPlot++ (Samson, Lord & Makarenkov, 2022) with 60 bp window size, 30 bp step, 33% gap string, and Kimura-2-parameter distances.

### Phylogenetic analyses

The phylogenetic analysis with rDNA genes included both 18S and 28S, which were aligned separately using MAFFT v7.505, with a global alignment algorithm. After removing ambiguously aligned positions with Gblock v0.91b (Castresana, 2000), individual MSAs were concatenated into a single nucleotide partition. The mtDNA data included all 14 PCGs, translated into amino acids, and both 16S and 12S rRNA genes. Both rDNA and mtDNA phylogenetic reconstructions were performed under the Maximum Likelihood (ML) criterion implemented in RAxML-NG v1.1.0 (Kozlov et al., 2019). We conducted 20 independent searches (10 from random starting trees and 10 from parsimony-based trees), retaining the best-scoring topology. Data partitions were analysed with the GTR+G10+FO model for nucleotides (rDNA and mtDNA) and the mtZOA+G10+FO model for amino acids (mtDNA). Branch lengths were estimated independently for each partition (unlinked), and branch support was assessed through 1,000 rapid bootstrap replicates using the Transfer Bootstrap Expectation (TBE) metric, or until convergence was reached, in increments of 500 replicates. For both analyses, we included all available Poecilosclerida species with complete or nearly complete 18S and 28S sequences (Table  S2) and mitogenomes (Table S1). We also used in both analyses *Halichondria okadai* (rDNA: AB511881; mtDNA: NC_037391) as the sole outgroup to optimize ingroup evolutionary rate estimation. This approach was justified by the well-established monophyly of Poecilosclerida (*e.g.*, Redmond et al., 2013; Thacker et al., 2013; Lavrov et al., 2023), which was further supported by preliminary analyses (data not shown), which also prompted the exclusion of *Crambe crambe* mtDNA due to its disproportional divergence.

## Results and Discussion

### Genome skimming

All samples were successfully sequenced with both high read counts (5.85 ± 1.34 million reads), given the aimed coverage, and average quality (24.6 ± 0.7 Q Score) (Table 2). Genome skimming from selected poecilosclerids exhibited a low proportion of low-entropy reads (29.1 ± 14.5‰) and minimal exogenous DNA in their metagenomes (31.8 ± 20.2‰), depicted by taxonomic classification of reads against bacteria, archaea, fungi, protists, and viruses references (Figs. 2A–2B; Table S3). However, reads mapped against genomes currently available for the order comprised solely 4.0–57.2‰ of the same datasets, evidencing that the organismal source of most of the reads has yet to be determined. Despite the low proportion of reads mapping to mtDNA (0.48 ± 0.57‰) and rDNA (1.19 ± 0.94‰) references, we were able to assemble complete mitochondrial genomes for four species and 45S rDNA clusters for all samples but one, which presented relatively high coverage (7–29× and 31–300×, respectively). The proportion of mtDNA and rDNA mapped reads greatly varied among poecilosclerids, which may reflect a variety of factors, from biases in DNA purification method to variation in holobiont structuring. Preliminary analysis on metagenome composition (Figs. 2C–2D) showed no association with either phylogenetic positioning (PERMANOVA: *F* = 2.252, df = 3, *R*^2^ = 0.2938, *p* = 0.0567) or biogeographic distribution (PERMANOVA: *F* = 1.705, df = 5, *R*^2^ = 0.3706, *p* = 0.1255), with associations at lower taxonomic and geographic scales likely masked by a wide disparity among samples.

**Table 2:** Raw and filtered read counts from genome skimmings in this study.

**#**	**Species**	**SRA**	**Raw reads**	**Av. Qual.**	**% GC**	Filtered reads (%)	**mtDNA**		**rDNA**		gDNA^*^
							**‰ mapped**	**Length (DP)**	**‰ mapped**	**Length (DP)**	**‰ mapped**
Ape	*Asbestopluma pennatula*	SRR36636196	7,241,578	24.5	45.6	6,457,370 (89.2)	0.675	–	2.389	7,015 (173×)	27.323
Cgr	*Chondrocladia grandis*	SRR36636195	5,781,250	22.5	44.1	4,903,032 (84.8)	1.207	–	0.836	6,831 (65×)	23.636
Cge	*Cladorhiza gelida*	SRR36636189	6,020,668	24.4	41.9	5,414,958 (89.9)	2.165	–	0.998	6,620 (66×)	29.292
CMsp	*Clathria* (*Microciona*) sp.	SRR36636188	3,494,456	24.2	45.0	2,845,596 (81.4)	0.434	18,725 (8×)	2.168	6,484 (121×)	16.323
Dan	*Desmapsamma anchorata*	SRR36636187	6,997,264	25.4	43.7	6,389,304 (91.3)	0.707	19,099 (29×)	0.616	6,687 (73×)	7.838
Dme	*Diacarnus megaspinorhabdosa*	SRR36636186	3,411,488	24.8	59.4	2,669,944 (78.3)	0.073	–	0.094	–	51.835
Gse	*Guitarra sepia*	SRR36636185	7,199,842	23.9	40.2	6,669,294 (92.6)	0.544	–	2.448	6,943 (300×)	57.172
Hfa	*Hamacantha falcula*	SRR36636184	4,572,116	24.4	48.0	3,840,932 (84.0)	0.040	–	1.244	6,308 (59×)	6.568
Lbi	*Latrunculia biformis*	SRR36636183	6,393,970	24.9	44.3	5,809,640 (90.9)	0.741	19,632 (26×)	1.194	6,687 (118×)	26.817
Lis	*Lissodendoryx isodictyalis*	SRR36636182	7,088,014	25.3	54.0	6,250,274 (88.2)	0.034	–	0.653	6,605 (73×)	3.976
MGsp	*Mycale* (*Grapelia*) sp.	SRR36636194	6,084,384	25.2	37.9	5,284,742 (86.9)	0.261	–	0.601	6,546 (60×)	8.080
Mdi	*Mycale* (*N.*) *diversisigmata*	SRR36636193	7,734,498	25.3	39.5	7,398,060 (95.7)	0.498	–	0.974	6,840 (122×)	13.093
Ntu	*Neopodospongia tupecomareni*	SRR36636192	5,414,768	24.8	47.6	4,744,152 (87.6)	0.199	19,983 (7×)	0.357	6,128 (31×)	9.708
PBsp	*Phlyctaenopora* (*Barbozia*) sp.	SRR36636191	4,950,442	25.2	52.5	4,236,938 (85.6)	0.032	–	1.087	6,661 (80×)	14.640
Udi	*Ulosa digitata*	SRR36636190	4,498,326	24.4	42.7	3,617,570 (80.4)	0.045	–	3.361	6,489 (232×)	22.837

**Notes.**

Permillage (‰) of mapped reads (in proportion to filtered reads) and averaged coverage onto assembled mitogenomes (mtDNA) and 45S ribosomal DNA clusters (rDNA), and onto the genomes (gDNA) of *Crambe crambe* (GCF_963924555), *Diacarnus erythraeanus* (GCF_964016965), and *Lycopodina hypogea* (GCF_963969325). Length (in bp) and average depth (DP) of contigs from complete (or nearly complete) sequences only.

**Figure 2:**
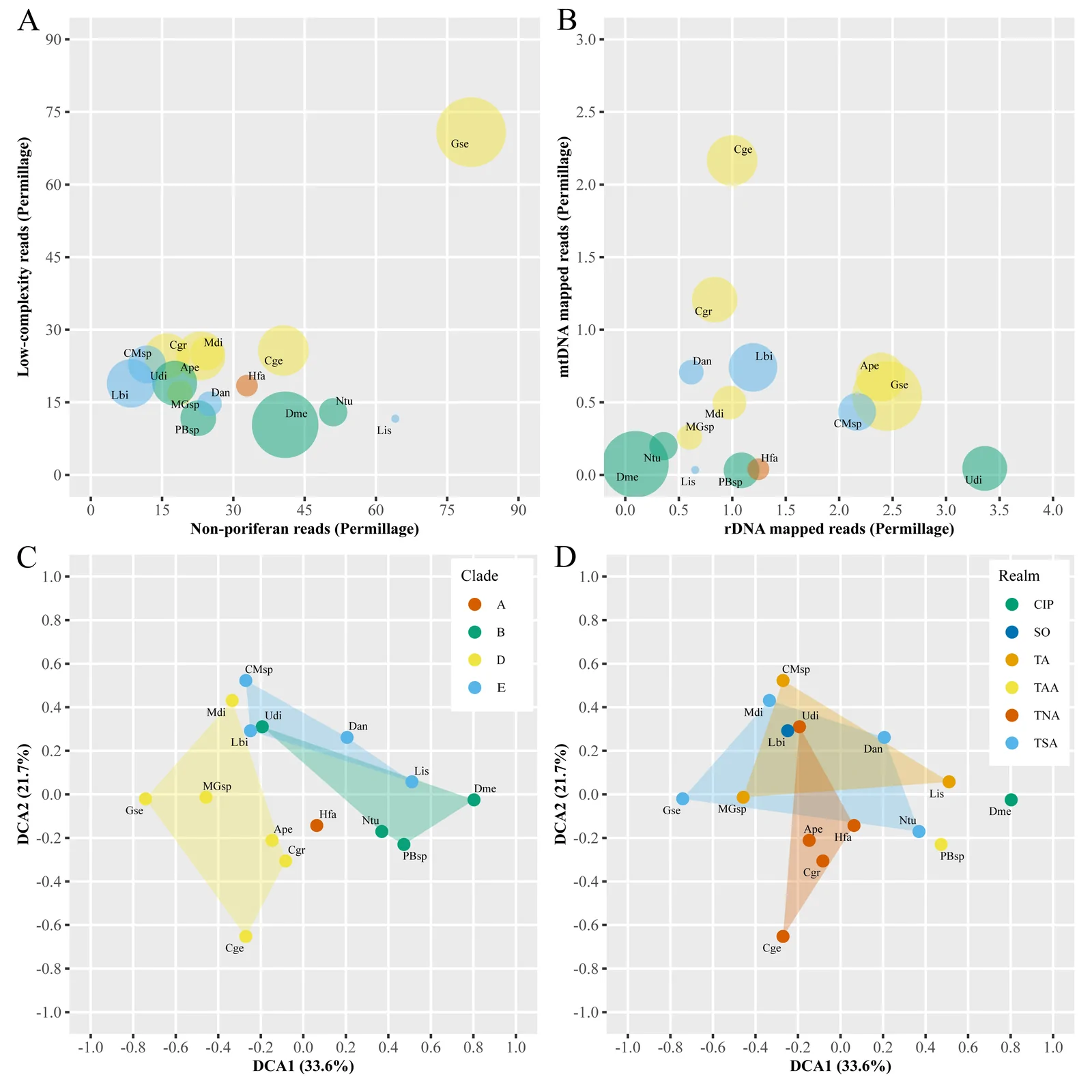
Proportion of reads mapped to different sources (A–B) and associated metagenome composition (C–D) in Poecilosclerida genome skimming. (A–B) Bubbles are scaled to the proportion of total metagenomic reads mapped to Poecilosclerida genomic references (gDNA; see Table 2). (A) Non-poriferan sources regarding results from Kraken2. (C–D) Detrended correspondence analysis based on proportion of reads from non-poriferan sources (Table S3). (C) Clade assignments based on rDNA phylogenetic tree (Fig. 3). (D) Biogeographic realms from sampled localities (Table 1) based on Spalding et al. (2007): CIP, Central Indo-Pacific; SO, Southern Ocean; TA, Tropical Atlantic; TAA, Temperate Australasia; TNA, Temperate Northern Atlantic; TSA, Temperate South America. Species abbreviations in Table 1.

**Figure 3:**
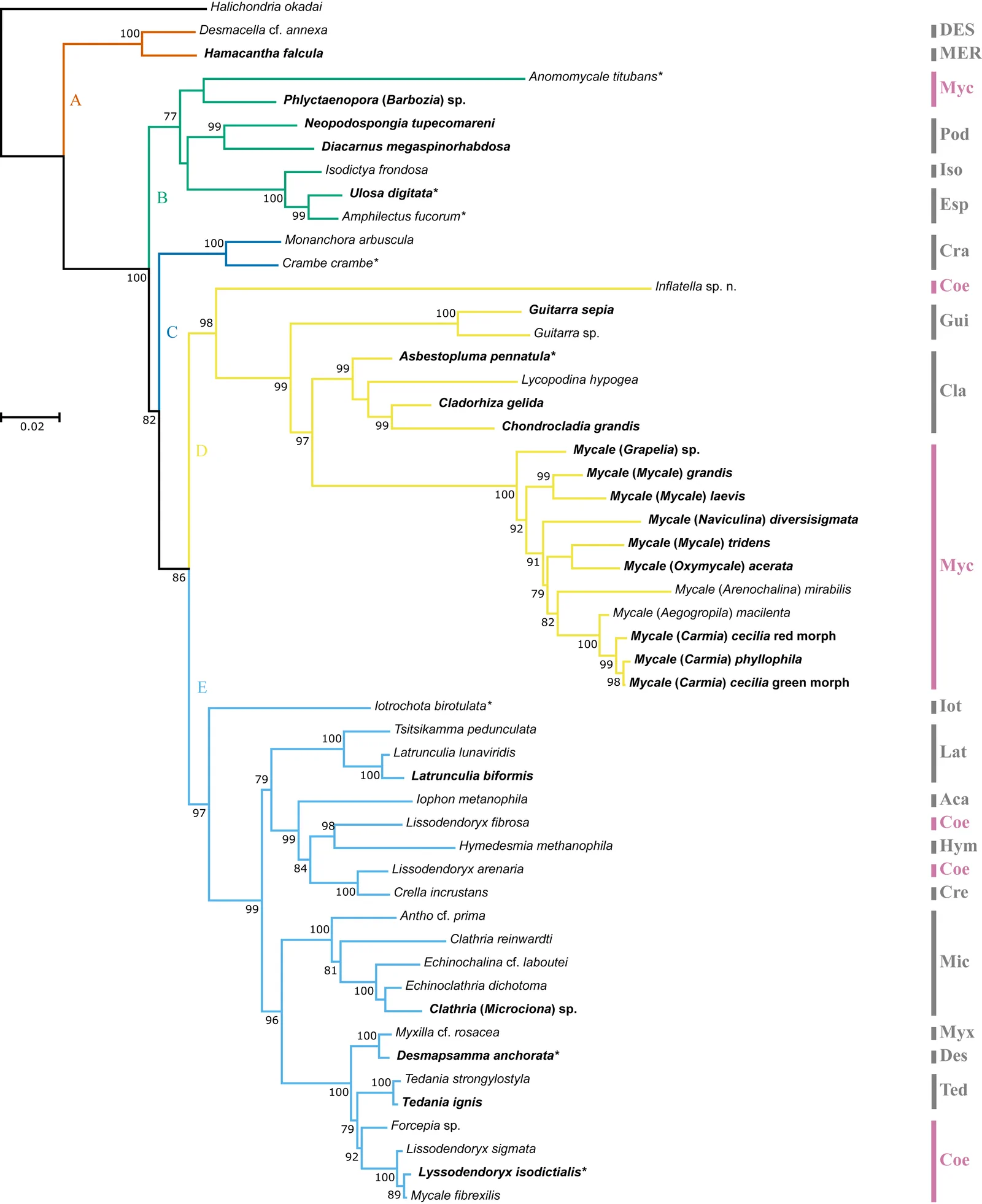
Phylogenetic reconstruction of Poecilosclerida rDNA cluster using nucleotide (18S and 28S) data. The maximum likelihood tree with the best score (*lnL* =  − 28126.676709) is shown, with bootstrap values indicated at nodes. Major clades (A–E) are indicated. Familial allocations are indicated, with polyphyletisms indicated in reddish. Type species of genera indicated by an asterisk (*). Newly generated sequences are highlighted in bold; accession numbers are provided in Table S2.

We were not able to retrieve enough rDNA data from *Diacarnus megaspinorhabdosa* to assemble meaningful contigs to be used in posterior analyses. In addition, all samples for which we failed to build complete mitochondrial genomes returned varying levels of partial sequences for mtDNA genes (https://doi.org/10.6084/m9.figshare.30742643), which were included in phylogenetic reconstructions accordingly. Specimens without complete mitogenomes assembled fall into two groups, one with very low read counts (136–196) and one with relatively high read counts (1,378–11,726) in the mapped dataset of mtDNA. Although the low read counts may explain our inability to assemble mtDNA from some of these skimmings (*e.g.*, *Diacarnus megaspinorhabdosa* and *Ulosa digitata*), the same is unlikely the case for most specimens with higher read counts, including Cladorhizidae, Guitarridae, and Mycalidae spp. For instance, *Chondrocladia grandis* and *Cladorhiza gelida* exhibited higher mtDNA mapped read counts in contrast to rDNA, but unlike mtDNA, we successfully assembled full rDNA sequences with >60 × coverage from these samples. Previous studies already highlighted a Mycalidae species, *viz.*, *Mycale escarlatei*, that might present an unusual mtDNA structure, including the possibility of multi-partite chromosomes (Lavrov & Pett, 2016). Also, the mtDNA of a Cladorhizidae species, *Lycopodina hypogea*, was recently described as presenting an unusual organization, with several rearrangements and putative small circular extrachromosomes (De Paula et al., 2024). In addition, the former poecilosclerids *Desmacella informis* (OM729615) and *Hamacantha johnsoni* (OM729619), currently allocated in their own orders, also present extensive rearrangements in their mitogenomes, as inferred from available partial sequences (Lavrov et al., 2023). Assembly of mtDNA mapped reads from those specimens recovered several truncated contigs that likely comprise genomic rearrangements and/or duplicated regions, as illustrated in Fig. S1. Thus, particular groups within Poecilosclerida *lato sensu* present mitochondrial genomes that, in contrast with the conservative structure observed in other lineages, are seemingly hyperrearranged. Our attempts to assemble the mitochondrial genome of *Mycale lingua* from PacBio HiFi data (ERR14835532), that has been recently made available by the Wellcome Sanger Institute, partially corroborate this hyperrearranged hypothesis, in which 1–15 kb long reads seem to contain mtDNA sequences arranged in several rearrangements (Fig. S1). Resolving structurally complex regions, such as those putatively involving duplications and rearrangements, is challenging with short-read sequencing alone due to limited read lengths (*e.g.*, ∼300 bp inserts in genome skimming). Even long-read sequencing (*e.g.*, ∼15 kb reads in *M. lingua*) may be insufficient to fully disentangle these regions if the repetitive or duplicated sequences exceed the read length. Therefore, ultra-long-read sequencing (*e.g.*, Oxford Nanopore Technologies, with reads exceeding 20 kb) may be essential for accurate mitogenome assembly in these divergent lineages.

### Phylogenetic analyses

Phylogenetic reconstruction based on rDNA data included both 18S and 28S sequences. After excluding 23% of original positions due to ambiguities, the final alignment comprised 4,698 nucleotides, of which 5.7% were gaps and 76.3% were invariant sites. The phylogenetic reconstructions took into account 112 free parameters (model + branch lengths) and bootstrap support was achieved after convergence at 3,550 replicates. The best-scoring, rDNA-based tree (Fig. 3) shows five major clades A–E comprising: A, Desmacellida + Merliida; B, Podospongiidae + Isodictyidae + Esperiopsidae + (non-*Mycale*) Mycalidae; C, Crambeidae; D, Guitarridae + Cladorhizidae + (*Mycale*) Mycalidae; E, Iotrochotidae + Latrunculiidae + Acarnidae + Coelosphaeridae + Hymedesmiidae + Crellidae + Microcionidae + Myxillidae + Desmacididae + Tedaniidae. As currently defined, we recovered substantial polyphyletism in families Coelosphaeridae and Mycalidae (to a lesser extent), which requires additional data and revisions. Although *Anomomycale titubans* is currently accepted as *Mycale* (*Anomomycale*) *titubans*, its phylogenetic positioning has long been proved unrelated to Mycalidae s.s. (Thacker et al., 2013). The elevation of *Anomomycale* to full generic status is pending, a change that may also necessitate the creation of a new family. The positioning of *Phlyctaenopora*, assessed for the first time here, nests it close to Podospongiidae and Esperiopsidae spp. Together, *Phlyctaenopora* and *Anomomycale* comprise a pseudo-Mycalidae clade, whose lack of support is likely due to heterogeneity in substitution rates. However, despite the polyphyletism of Mycalidae (Hajdu et al., 2013; Redmond et al., 2013), *Mycale* rests monophyletic after our analyses. Regarding its subgenera, *M.* (*Carmia*), with three species included in the analyses, surprisingly turned out monophyletic. Given the subgenus’ simpler skeletal architecture in contrast to other subgenera, monophyly was unexpected. The 18S phylogeny constructed by Redmond et al. (2013) retrieved unclear relationships within *Mycale* due to the low support of many nodes. In particular, it is unclear how *M.* (*Carmia*) relates to *M.* (*Aegogropila*) and *M.* (*Zygomycale*). These three subgenera nested in a highly supported clade in the quoted study, albeit both latter subgenera represented by a single species each, respectively *M.* (*Aegogropila*) *rotalis* and M. (*Zygomycale*) *angulosa*. The Coelosphaeridae case is complicated further by the polyphyletism of *Lissodendoryx*, which has also been previously discussed (*e.g.*, Redmond et al., 2013; Thacker et al., 2013). Although the monophyletism of Microcionidae was recovered, *Clathria* comprises a paraphyletic taxon also pending revision, as previously highlighted elsewhere (Redmond et al., 2013; Cruz-Barraza, Carballo & Aguilar-Camacho, 2023; Turner & Lonhart, 2023). Finally, we recovered *Inflatella* sp. n. as basal to Clade D, contrasting with previous phylogenetic studies that used 18S (Redmond et al., 2013) or 28S (Thacker et al., 2013) sequences independently. However, according to these authors, *Inflatella pellicula* (KC902151), the type species of the genus, is not closely related to *Inflatella* sp. n., and thus the latter must be re-examined prior to any further systematic conclusions.

In the mtDNA analysis, all 14 protein coding genes and both rRNA genes were included in the final alignment. The proportion of ambiguously aligned regions across individual PCG alignments was ca. 10% higher than in previous studies (De Paula et al., 2024; Leite, De Paula & Hajdu, 2024). This is likely due to the inclusion of partial mtDNA sequences recovered from skimmings. Contrastingly, the number of retained regions for *rnl* (67%) was higher. The final dataset included 7,265 positions, whose partitions presented 3,930 amino acids (gaps = 21.2%; inv. sites = 44.2%) and the DNA partition 3,335 nucleotides (gaps = 20.3%; inv. sites = 36.6%), totaling 93 free parameters. The best-scoring mtDNA tree (Fig. 4) conflicts in several ways with the rDNA topology. It clusters *Phlyctaenopora* and *Ulosa* in a long branch, likely due to the overall lack of mitogenomic data from these species. It places *Iotrochota* closer to clade D. It also places the subclade containing Tedaniidae and Desmacididae spp. more basally in clade E. However, in the rDNA tree, *Tedania* and *Desmapsamma*, representing both families, nest amidst many arcuate and anchorate chelae-bearing poecilosclerids (*viz.*, *Forcepia*, *Lissodendoryx*, *Myxilla*). These genera form a sister subclade to the Microcionidae clade (with *Antho*, *Clathria*, *Echinochalina*, and *Echinoclathria*). The former includes *Mycale fibrexilis* also, a misidentified sequence according to Hajdu et al. (2013). This misidentification keeps generating spurious results (*e.g.*, Cruz-Barraza, Carballo & Aguilar-Camacho, 2023). Both rDNA and mtDNA trees cluster Cladorhizidae and Mycalidae together, with Guitarridae basal to them. *Lissodendoryx* was retrieved polyphyletic in both trees, while *Hymedesmia* and *Phorbas* were polyphyletic in the tree based on mitogenomes (Fig. 4), corroborating earlier findings (Redmond et al., 2013; Cruz-Barraza, Carballo & Aguilar-Camacho, 2023; Turner & Lonhart, 2023), now including the type species of *Lissodendoryx*, *viz. L. isodictyalis*.

**Figure 4:**
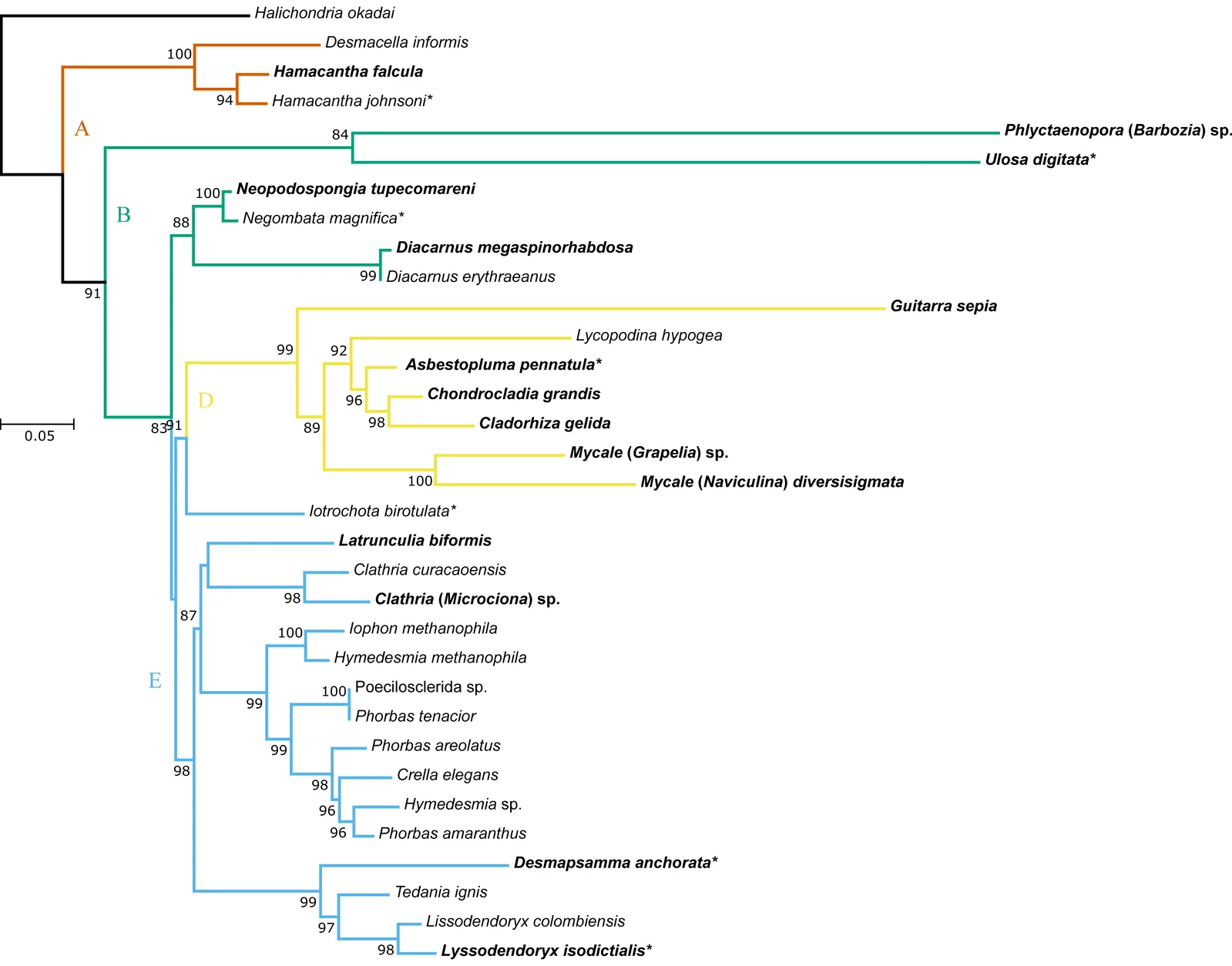
Phylogenetic reconstruction of Poecilosclerida mitogenomes using amino acid (14 PCGs) and nucleotide (16S and 12S rRNA) data. The maximum likelihood tree with the best score (*lnL* =  − 81105.689921) is shown, with bootstrap values indicated at nodes. Major clades (A–E) correspond to the rDNA topology (Fig. 3). Type species of genera indicated by an asterisk (*). Newly generated sequences are highlighted in bold; accession numbers are provided in Table S1.

### Mitogenome structure and composition

We were able to recover complete mitochondrial genome sequences from four specimens, *Clathria* (*Microciona*) sp., 18,725 bp long (GenBank accession PX693172; Tables S4, S5), *Desmapsamma anchorata*, 19,099 bp (PX693173; S6, S7), *Latrunculia biformis*, 19,630 bp (PX693174; S8, S9), and *Neopodospongia tupecomareni*, 19,983 bp (PX693175; S10, S11). All mitogenomes include 14 PCGs, a varying number of tRNAs (22–25), and two rRNAs (rnl/16S and rns/12S) genes (Fig. 5). These mitogenomes lack an organized control region and include the *atp9* gene, as usual in sponges and in contrast to bilaterians (Lavrov & Pett, 2016). We observed several gene overlaps between PCGs and tRNAs, with consistent patterns across species. A 10 nt overlap between *cox1* and *trnS(uga)* occurs in all four mitogenomes, as well as a 3 nt overlap between *trnE(uuc)* and *nd6*. The *atp8* and *atp6* genes show an 11 nt overlap in *C.* (*Microciona*) sp. and *L. biformis* but only 7 nt in *N. tupecomareni*. The *cox2* and *trnF(gaa)* genes overlap by 10 nt in most species, though they are separated by an extensive (>160 nt) intergenic region in *C.* (*Microciona*) sp. These conserved overlapping positions between PCG and tRNAs further suggest a role of the latter in gene rearrangements (see below). The base composition of these mtDNAs varied within close ranges (*G* = 21.5–23.4%, *A* = 28.7–30.7%, *T* = 36.4–38.0%, *C* = 9.4–11.5% (Tables S5, S7, S9, S11). All species’ AT-skews were negative (−0.13 to −0.10) while their GC-skews were positive (0.34 to 0.40), thus consisting of a higher abundance of Ts and Gs.

**Figure 5:**
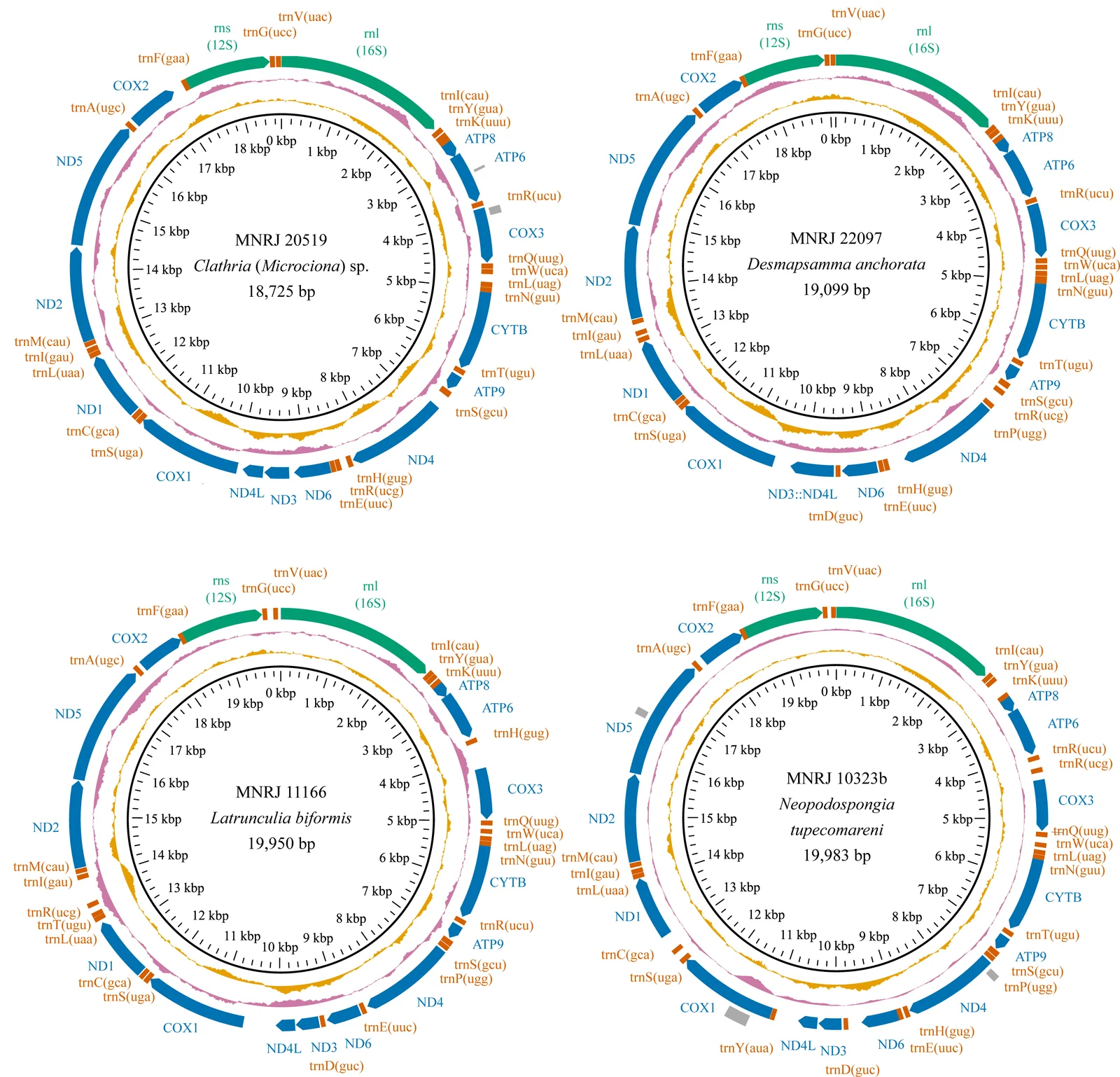
Mitochondrial genome maps generated here. Coding genes in colors: protein (blue), ribosomal RNA (green), transfer RNA (red). GC-content (pink) and GC-skew (orange) plots are shown. Grey boxes are missing data from assemblies.

#### PCGs and codon usage

The fourteen PCGs covered 61.7–65.6% of the mitochondrial genomes, encoding in total 4,076–4,094 amino acids (Tables S4, S6, S8, S10). The proteins encoded include seven NADH dehydrogenases (ND1–ND6, and ND4L, in complex I), one cytochrome b (CYTB, in complex III), three cytochrome c oxidases (COX1-COX3, in complex IV), and three ATP synthases (ATP6, ATP8 and ATP9, in complex V). In *D. anchorata*, the *nd3* and *nd4L* genes were fused in-frame into a single open reading frame (ORF), putatively encoding an ND3::ND4L protein. While ND3 and ND4L subunits were previously considered non-critical for mitochondrial complex I function, given their absence in some algal mitochondrial genomes (Cardol et al., 2006) and their lower prevalence of damaging mutations in humans (Gonzalez-Halphen et al., 2011), recent evidence suggests another possibility. A hydration bridge between ND3 and ND4L may promote proton influx through ND6, driven by ND4 and ND5 pumps (Parey et al., 2021). Although the functional impact of this fusion remains unclear, its occurrence aligns with the spatial proximity of ND3 and ND4L within complex I, and provided the fusion preserves viable interactions and conformational dynamics, we regard this gene fusion as potentially functional. The smallest PCG, *atp8*, ranges from 210 bp (69 aa), in *D. anchorata*, to 231 bp (76 aa) in *Clathria* sp., while the largest, *nd5*, has 1,878 bp (625 aa) in the latter, and 1,893 bp (630 aa) in the remaining mtDNAs. As usual for sponges, all genes lie on the heavy (H) strand. Most of the genes in all mitogenomes use the typical AUG start codon, with the exception of: *atp8* and *cox2* in *D. anchorata*; *cytb*, *atp9*, and *cox2* in *L. biformis*; and *nd6*, *nd5*, and *cox2* in *N. tupecomareni*, which use GUG as initiation site. In the mitogenome of *Clathria* (*Microciona*) sp., in addition to the GUG start codon in the *nd4*, *nd6*, *cox1*, and *cox2* genes, we found the codon UUG as the first in-frame codon of the *cytb* gene. Another unusual initiation codon, GUA, was observed in the *nd6* gene of the *D. anchorata* mitogenome. Non-canonical start codons are well-documented in the mitochondrial genomes of animals. A widely known, multi-study case is the starting codon UCG proposed for the *cox1* gene from mitogenomes of *Drosophila* and *Anopheles* species (Krzywinski, Grushko & Besansky, 2006; Beckenbach & Stewart, 2009; Hao et al., 2017). In glass sponges, the AUA codon had been reported (without further discussion) as the start site of the *atp9* gene in the mitogenome of *Docosaccus maculatus* (Kahn & Geller, 2018). On the other hand, the stop codons UAA and UAG were widely used across the mitogenomes, with the former being more prevalent in mtDNA of *Clathria* sp. and *D. anchorata* (nine and eight instances) than in *L. biformis* and *N. tupecomareni* (five instances each). All 14 PCGs followed the negative AT-skew and positive GC-skew pattern from the overall mitogenome. The codon degeneracy pattern for the PCGs encoded in all four mitogenomes showed both two- and four-fold degenerate codons are biased toward the use of codons with U (against C) in the third position (Fig. S2). The amino acid leucine used the codon from the mitochondrially encoded tRNA${}_{UAA}^{Leu}$ disproportionately more than all other amino acids, which had similar or significant lower usage of codons from their associated mtDNA tRNAs. Amino acid usage across PCGs from the newly described mitogenomes revealed leucine (L; 14.4–15.2%) and isoleucine (I; 10.6–12%) as the most used ones, and histidine (H; 1.2–1.7%) and cysteine (C; 1.0–1.4%) as the least used amino acids. The amino acid composition of Poecilosclerida mitogenomes (Fig. 6) showed strong phylogenetic clustering (PERMANOVA: *F* = 5.484, df = 4, *R*^2^ = 0.4576, *p* = 0.0001), with isoleucine (I: 11.4%), glycine (G: 10.7%), and valine (V: 8.0%) contributing most to the observed variation.

**Figure 6:**
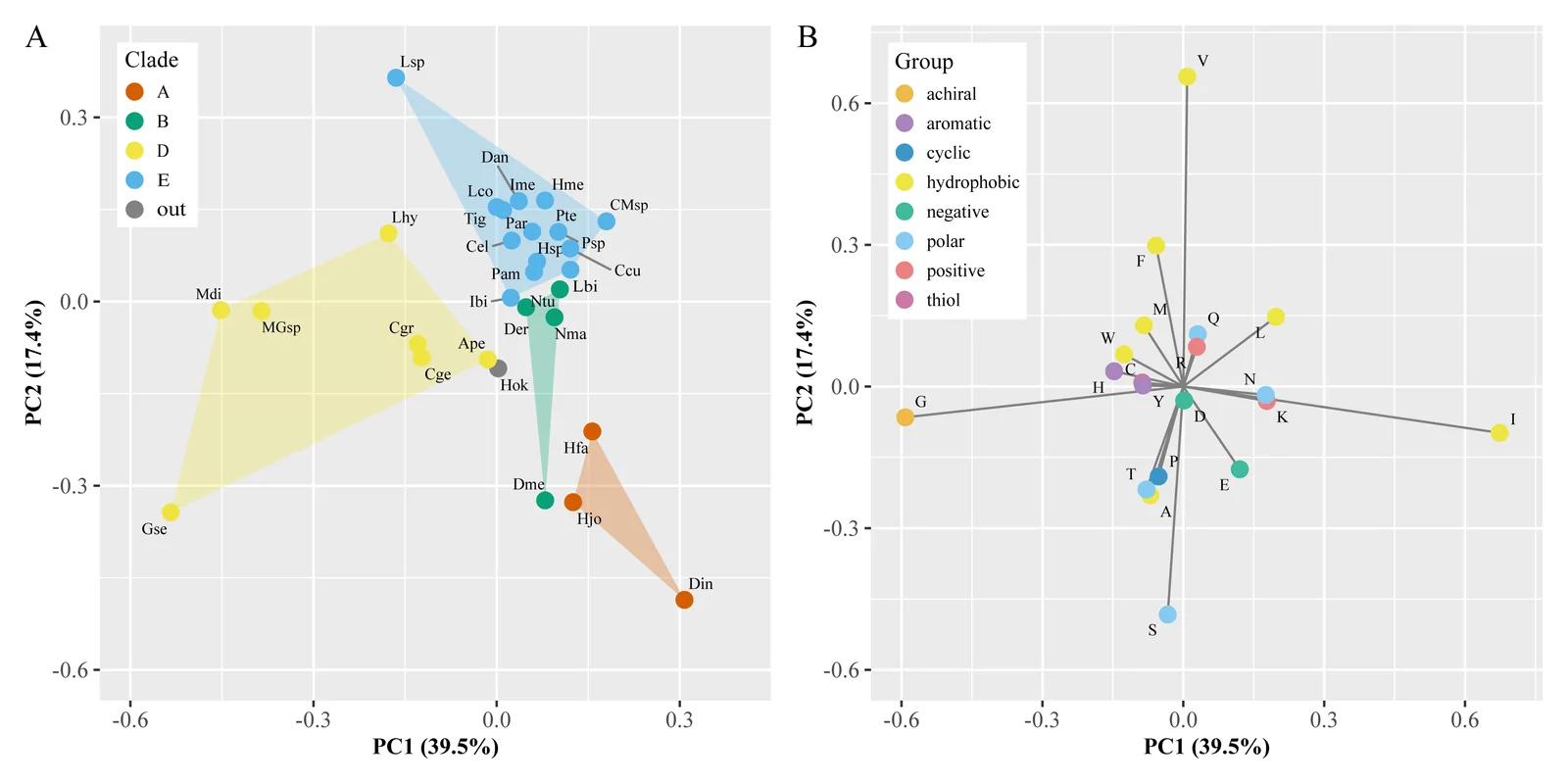
Amino acid composition across Poecilosclerida mitogenomes. Principal component analysis based on amino acid proportion in concatenated aligned genes used in phylogenetic reconstruction. *Phlyctaenopora* (*Barbozia*) sp. and *Ulosa digitata* were excluded due to lack or low amino acid composition in the alignment. (A) Species clade assignments based on rDNA phylogenetic tree (Fig. 3). (B) Amino acids biochemical properties according to groups. Species abbreviations in Table 1 and Table S1.

#### Ribosomal and transfer RNAs

The large subunit (*rnl*; 16S rRNA) and small subunit (*rns*; 12S rRNA) ribosomal RNA genes from the new mitochondrial genomes are both located on the H-strand, ranging 2,248–2,572 bp and 1,206–1,257 bp, respectively. For all species, AT and GC skews were similar, with *rnl* showing less variation across species, and more congruence with overall mtDNA skewness, than *rns* genes, indicating a higher concentration of guanine nucleotides in those rRNAs. Conserved sequences at both 5′ and 3′ ends of rRNA genes were consistently observed across all examined mtDNAs. While these conserved regions facilitated initial gene boundary delineation, comprehensive secondary structure analysis and identification of cleavage sites involved in processing these transcripts from larger precursors are still pending. Thus, we highlight the need for a definitive characterization of *rnl* and *rns* gene boundaries across all Poecilosclerida mitogenomes once additional evidence emerges.

The mtDNAs of *D. anchorata* and *N. tupecomareni* match the usual 24–25 tRNA genes found in other poecilosclerids, in contrast to *Clathria* sp. and *L. biformis*, with 22 and 23 tRNAs respectively. Losses of tRNA genes in these mitogenomes are in agreement with other reports in related species, with the exception of *trnP(ugg)* lost in *Clathria* sp. and *trnR(ucg)* in *L. biformis*. All tRNAs are encoded on the H-strand and present a typical clover structure (data not shown). Transfer RNA genes constitute 8.6–9.2% of these mitogenomes, with lengths ranging from 68 to 87 bp. A notable exception is the *trnN(guu)* gene in *D. anchorata*, which contains a 39 nt “intron” derived from a direct duplication of the immediate upstream region (Fig. S3). The presence of this insert raises questions about the functionality of tRNA${}_{GUU}^{Asn}$, as its retention would disrupt the canonical secondary structure of the tRNA (with an elongated arm and altered anticodon loop sequence). Despite this apparently compromised structure of the *D. anchorata trnN(guu)* gene, we refrain from classifying it as a pseudogene without further evidence. Overall, tRNA content among Poecilosclerida mitogenomes seems to present a few phylogenetic constraints and several lineage-specific changes. For instance, the *trnY(gua)* gene was independently lost in two distinct lineages: in Esperiopsidae, clade B, and at the basis of clade E. This is inferred by the presence of *trnY(gua)* in almost all other heterosclemorph sponges (Lavrov et al., 2023), in podospongiids (here), *Crambe crambe*, and *Lycopodina hypogea* (De Paula et al., 2024), from clades B, C, and D, respectively. Another example would be the apparent loss of *trnD(guc)* in the Microcionidae subclade (in clade E). All other cases of tRNA loss are restricted to specific lineages, as presented above, pending the description of additional poecilosclerid mtDNAs from further lineages.

#### Intergenic regions and gene rearrangements

Intergenic regions (IGRs) comprise a higher proportion of the mitochondrial genomes of *L. biformis* (10.9%) and *N. tupecomareni* (10.3%), than from *Clathria* sp. (6.0%) and *D. anchorata* (6.8%). The overall base composition in IGRs was similar among species (*G* = 21.4–24.1%, *A* = 32.4–42.5%, *T* = 31.6–37.3%, *C* = 4.5–12.1%), notably in guanine and thymine contents. Conversely, we observed higher variation on GC-skew values among species (0.33–0.72) than AT-skews (−0.04–0.13). The 46 nt IGR between *nd1* and *trnL(uaa)* in *L. biformis* mtDNA (spanning positions 13,303–13,348 bp) contains a 42 nt sequence with a single mismatch against its own *rns* gene (initially identified *via* a BLAST hit against *Negombata magnifica*). This observation suggests an ancestral rearrangement involving genes not typically adjacent to *rns*, given their conserved positioning in related poecilosclerid mitogenomes. All IGRs over 30 bp in *N. tupecomareni* produced BLAST hits against homologous regions in *Negombata magnifica* mtDNA, demonstrating conserved gene order as well as sequence similarity in intergenic regions between these podospongiid species. The mtDNA of *Clathria curacaoensis* (OM729636) is absent from the BLAST core nucleotide database, likely owing to its UNVERIFIED status. Consequently, homologies between its IGRs and those of *Clathria* sp. were evaluated *via* global alignment. As observed previously, both microcionid sponges share identical IGR regions, with all sequences ≥30 bp exhibiting homology between the mitogenomes of *Clathria* sp. and *C. curacaoensis*. Most of these aligned regions contained indels of 4–8%, though a few displayed longer indels spanning 37–40%, reflecting substantial heterogeneity and divergent evolution in these sequences. Although multiple short translatable segments were extracted from intergenic regions across all species examined, comparative analysis against established protein databases confirmed these sequences lack functional significance.

Evolution through gene rearrangements in the mitochondrial DNA of Poecilosclerida seems to diverge greatly among lineages (Fig. 7). While clade B and clade E sponges present a relatively conserved gene order, species from clades A and C have putatively hyperrearranged mtDNAs. This excessive gene rearrangement is evident in the complete mitochondrial DNA of *Lycopodina hypogea* (De Paula et al., 2024) and the partial reports from *Desmacella informis* and *Hamacantha johnsoni* (Lavrov et al., 2023). This also finds some echo here due to the several broken edges in the assembly of contigs from clade C species, despite the high coverage of mtDNA-mapped reads, as argued before (Fig. S1). Although we initially blamed short read sequencing for the lack of success in assembling clade C mitogenomes, we recently had access to long read sequencing from *Mycale lingua* (ERR14835532, confirmed as a clade C species by phylogenetic analysis, unpub. data), which also failed to assemble a mtDNA without recurring to multiple within read rearrangements (TS de Paula, 2025, personal observations). We anticipate that Nanopore sequencing data will help resolve current assembly challenges in clade A and C poecilosclerids, as the ultra-long reads could verify if their mtDNAs consist of (i) multiple highly rearranged chromosomes, or (ii) a single (or fewer) larger molecule containing hyperrearranged, duplicated regions. This latter hypothesis is supported by the exceptionally large (≥30 kb) mtDNA of *L. hypogea*, which also contains putative multipartite microDNAs. Since these small extrachromosomal circular DNAs could not be assembled using long read sequencing—putative chrM2 has 291 bp, including a functional copy of *nd4L* gene, absent in the nuclear DNA (De Paula et al., 2024)—a hybrid Nanopore-Illumina sequencing approach appears better suited for assessing intrinsic biological characteristics of these hyperrearranged mitogenomes, circumventing most of the technical limitations these sequencing platforms may present. On the other hand, the poecilosclerid conserved mitogenomes comprise mostly tRNA gene losses and rearrangements. Overall, we were able to track twenty organizational changes in available mtDNAs, but that of *Crambe crambe* (disregarded from this study), most of these restricted to specific lineages, and likely a consequence of the current low diversity representation. However, we highlight the frequent rearrangements involving *trnR(ucu)*. These positions seemingly changed five times along clade E evolution, assuming its adjacent position to *atp6* as the primitive state in podospongiids. Likely shifts occurred (i) closer to *rnl* in *Iotrochota birotulata*; (ii) closer to *cytb* in *L. biformis*; (iii) closer to *nd4* in microcionids; (iv) closer to *rns* in the *Hymedesmia*/*Phorbas* subclade; and (v) closer to *atp9* in the *Desmapsamma*/*Tedania* subclade. The atypical repositioning of *trnR(ucu)* stands in marked contrast to the (somewhat) conserved positions of other tRNAs, suggesting the existence of an unidentified molecular process. Notably, tRNAs have been demonstrated as targets of Ty1 LTR-retrotransposons in yeast and mold mitochondria (Winckler, Szafranski & Glöckner, 2005; Mularoni et al., 2012). Subsequent research established that 3′-end tRNA fragments complement long terminal repeats (LTRs) in retroelements, serving dual roles as primers for retrotransposon reverse transcription and as mediators of RNA interference that suppress their mobility (Schorn & Martienssen, 2018). The notable prevalence of mobile elements in the metagenome of *L. hypogea* raised questions on their role in the evolution of its hyperrearranged mtDNA (De Paula et al., 2024). Discussions on gene order evolution in demosponge mitogenomes typically rely on protein-coding and rRNA genes, since tRNA dynamics are either overlooked or dismissed by lack of informative data (Lavrov, Wang & Kelly, 2008; Lavrov et al., 2023). In the absence of direct evidence, the mobility of tRNAs, whether in conserved or hyperrearranged poecilosclerid mtDNAs, remains speculative. However, established links between mobile elements and mitochondrial evolution in fungi (*e.g.*, El Hage et al., 2014; Wu & Hao, 2015) provide a compelling rationale for parallel investigations in sponges, in which tRNA genes may play a major role in mitogenomic rearrangements. For instance, in an evolutionary context driven by element mobility, the complete loss of tRNAs in certain haplosclerid mitogenomes, currently seen as “disadvantageous in the long run” (Lavrov, Turner & Vicente, 2025), could represent adaptive short-term responses to escape parasitic DNA proliferation, outweighing potential long-term consequences. In striking contrast, clade D sponges, comprising cladorhizids, guitarrids, and mycalids, appear to maintain elevated evolutionary flexibility, given they represent ca. 6% of known demosponges, despite possessing putatively hyperrearranged mitogenomes.

**Figure 7:**
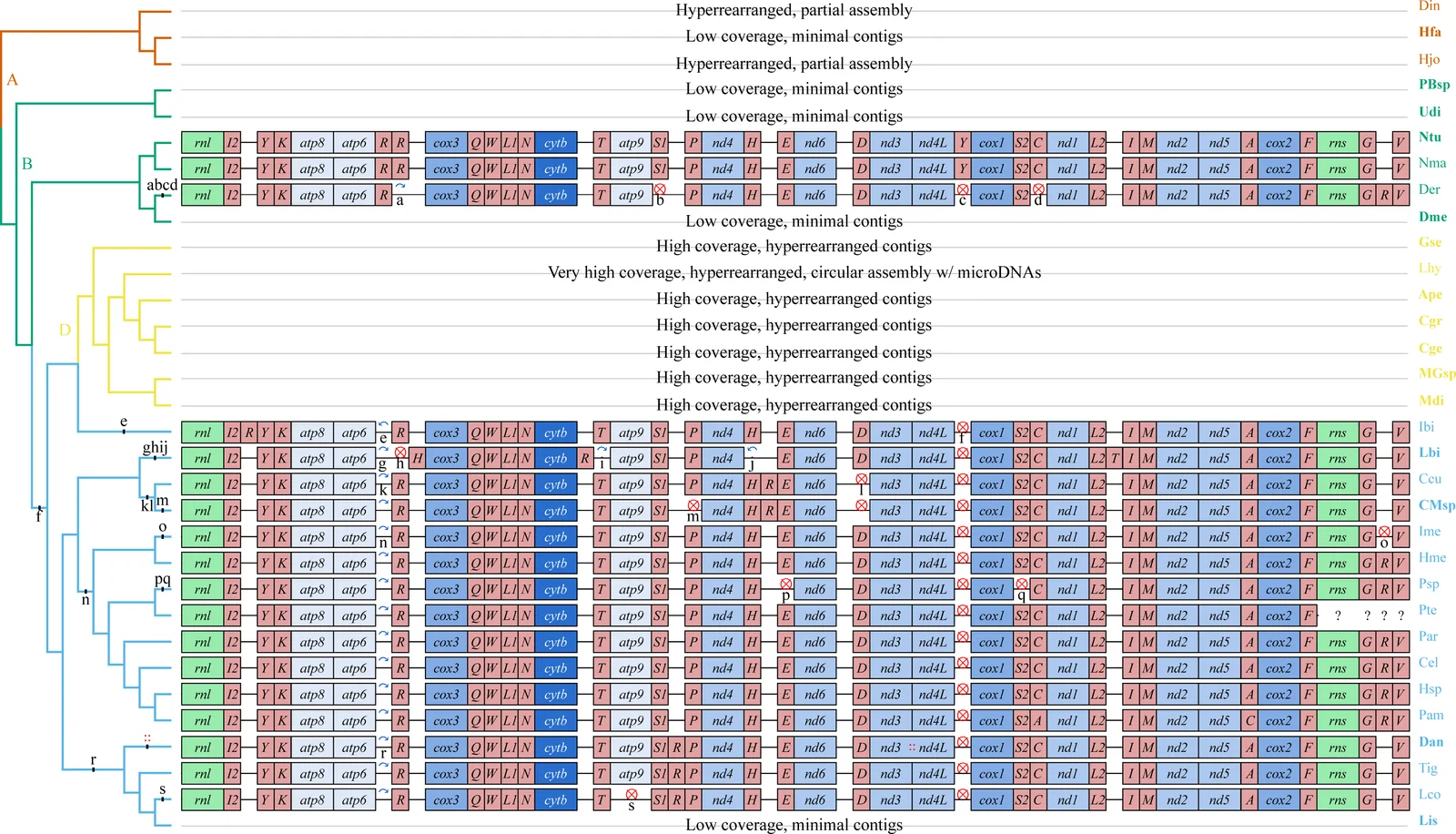
Rearrangements among Poecilosclerida mitochondrial genomes. Only conserved organizations are shown. Tree topology after the mtDNA ML reconstruction (Fig. 4). Symbols indicate loss (red cross), gene rearrangement upstream (blue left arrow) and downstream (blue right arrow), and missing data (?).

### Nuclear ribosomal DNA

Complete 45S nuclear rDNA clusters were successfully assembled from all examined poecilosclerid species, with the exception of *Chondrocladia grandis* (see below). These were supplemented by nine additional rDNA sequences obtained from published transcriptomic data (Table S2). The alignment of the entire 45S cluster revealed conserved blocks flanking all three rRNA genes, enabling precise boundary determination: **18S**, ranging 1,810–1,827 bp, exhibited highly conserved terminal motifs at both 5′-end (5′-GTT ACC TGG TTG ATC CTG CCA GTA GTC ATA-3′) and 3′-end (5′-TCC GTA GGT GAA CCT GCG GAA GGA TCA TTA-3′); **5.8S**, showing minimal length variation (155–156 bp), presented a conserved 5′-end (5′-AYA ACT TCT AAC GGT GGA CCC CTC GGC TCG TG-3′) and a variable 3′-end (5′-HHH CCD GRM GCA CGT CYG TCY GAG MGT YY-3′); and **28S**, spanning 3,633–3,861 bp, displayed moderately conserved termini at both ends (respectively, 5′-GRA CCT CRG ATC RGG CGA GRY YAC CCG CYG-3′ and 5′-TGA GAT TAA GCC TTD VHT GTC CTR MAG ATC-3′). Similarity patterns across the rRNA gene cluster were found to be comparable among all Poecilosclerida clades (Fig. 8). While ITS1 (67–351 bp) and ITS2 (184–296 bp) exhibited expected high sequence divergence (with numerous indels hindering comparisons), the D2 region of the 28S rRNA gene showed pronounced variability across all clades, further supporting its established utility for species delimitation (Erpenbeck et al., 2016). Region V9 (18S) was highly variable in all poecilosclerids, though less so in clade A, followed by region V2 by a wide margin. Variation in region D12 (28S) was also notably elevated, conversely, being the highest in clade A. Higher variation among clade C species was additionally observed in 28S regions D2–D6, in contrast to other poecilosclerids. *Asbestopluma pennatula*, similarly to *L. hypogea*, appears to present a rDNA cluster that includes the 5S rRNA gene, separated from 28S by an ITS3. In all other poecilosclerids examined, the 5S rRNA gene was recovered in a distinct contig, including the other cladorhizid species (TS de Paula, pers. obs.). Assembly of *C. grandis* rDNA cluster into a single contig failed likely due to a poly-G sequence (ranging 21–41 mer in reads) which appears to encompass the ITS1 region, with further evidence needed to solve its rRNA gene organization.

**Figure 8:**
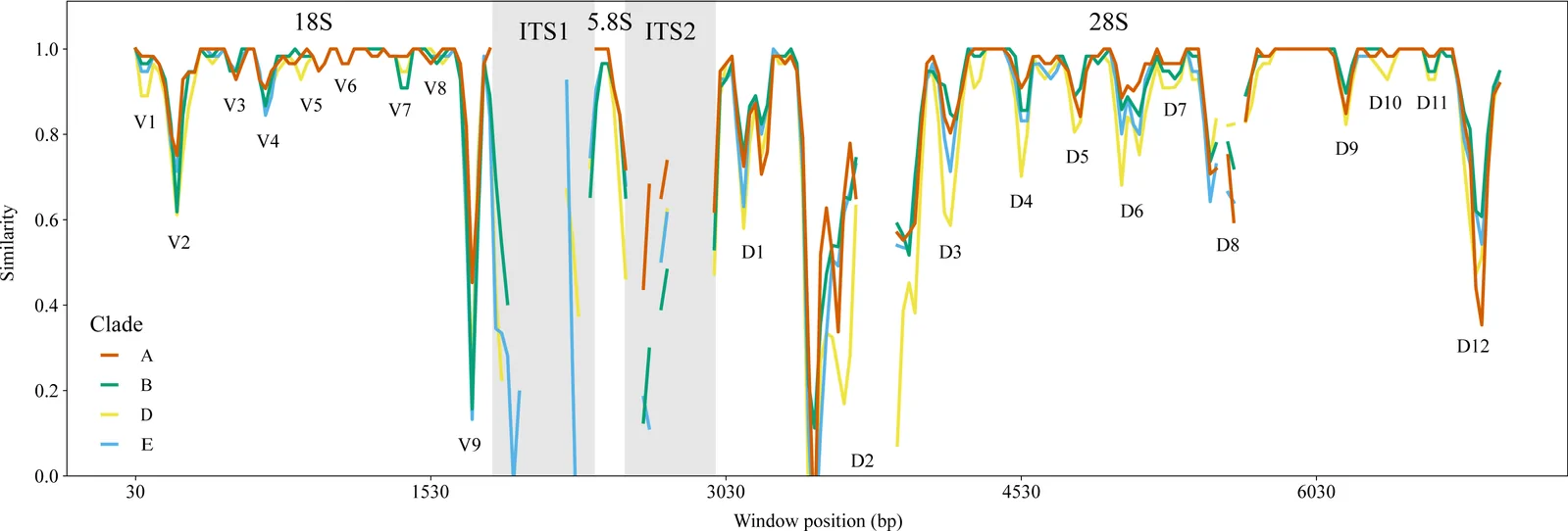
Similarity plot of 45S rDNA gene cluster among Poecilosclerida. Similarity values based on k2p genetic distances with a sliding window of 60 bp. Ribosomal RNA genes (18S, 5.8S and 28S) and internal transcribed spacers (ITS1 and ITS2) are indicated. Position of variable (V) and divergent (D) regions within major genes are shown. Clades according to rDNA phylogenetic tree (Fig. 3).

### Genome skimming as a tool for systematics and phylogenetics of sponges

Genome skimming has proved a highly efficient and cost-effective approach for Poecilosclerida phylogenetics, offering advantages over traditional methods while simultaneously capturing valuable metagenomic data. By sequencing total genomic DNA at low coverage (1–5×), this method recovered not only standard phylogenetic markers (18S and 28S rDNA and mtDNA genes), but also metagenomic sequences from associated microbial communities. This dual utility makes genome skimming particularly valuable for sponges, which host dense and taxonomically informative microbiomes (Easson & Thacker, 2014; Steinert et al., 2017; Sacristán-Soriano, Pérez Criado & Avila, 2020). While Sanger sequencing of individual markers typically costs USD 1–9 per reaction, assembling a comparable dataset for multiple loci across many samples becomes expensive. For example, for the same fifteen samples analysed here, solely sequencing (nearly) complete 18S and 28S genes *via* Sanger could cost up to USD 900 (assuming USD 5 per reaction, two and four fragments per gene, respectively, in both directions). In contrast, that would amount to six times the sequencing cost of our genome skimming on a NovaSeq 6000 Illumina platform, which allowed us to generate all these regions, in addition to mtDNA (with varying levels of coverage) and (some) metagenomic data. However, library preparation costs can reach up to USD 100 per sample, which far exceeds the amplification and purification costs required for Sanger sequencing. This is a major bottleneck for broad application of genome skimming in faunistic surveys, despite its potential to uncover a hidden diversity within well-established groups (*e.g.*, Turner et al., 2025). Since most of the (scarce) funds allocated to biodiversity and systematic studies are directed towards travel and accommodation services, this area would greatly benefit from improved and economically viable in-house methods that would allow for the broad application of next-generation sequencing (NGS) data (*e.g.*, Quail, Swerdlow & Turner, 2009; Glenn et al., 2019; Li et al., 2019; Zhao et al., 2020).

However, contrary to target methods, genome skimmings can be unreliable in generating specific loci data, especially when genomes widely vary among samples in size and/or complexity. Here, we failed to generate enough mtDNA reads from five species (*viz.*, *Diacarnus megaspinorhabdosa*, *Hamacantha falcula*, *Lissodendoryx isodictyalis*, *Phlyctaenopora* sp., and *Ulosa digitata*), likely hindering mitogenome assemblies (not including species with putative hyperrearranged mitogenomes). Preliminary (pilot) studies utilizing genome skimming across diverse taxonomic samples are essential to optimize sequencing depth, thereby retrieving aimed data with sufficient coverage and ensuring reproducible results. This process should involve assessing the suitability of DNA extraction methods, as even minor procedural adjustments, such as variations in centrifugation, can significantly alter mitochondrial DNA yield (Macher et al., 2018). Likewise, certain commercial kits may introduce biases in library preparation, sometimes resulting in the underrepresentation of mitochondrial sequences (Guo et al., 2009; Nacheva et al., 2017). Another factor to consider is the potential inherent variation in mitochondrial abundance across different sponge cell types, likely reflecting differences in metabolic demand. In human and murine tissues, for example, mtDNA copy number varies dramatically, up to 50-fold and 200-fold, respectively, between tissues with the lowest (blood or fat) and highest (heart) counts (Rath et al., 2024). Poriferan archeocytes and sclerocytes exhibit a notably higher mitochondrial density compared to choanocytes, for instance (Simpson, 1984). This cellular discrepancy may explain the exceptionally high proportion of mtDNA reads observed in the carnivorous *L. hypogea* (De Paula et al., 2024), which are rich in both archeocytes and sclerocytes (Le Goff et al., 2022; Baghdiguian et al., 2023). Conversely, library preparations from sponge tissues rich in cell types with low mitochondrial abundance could thus yield reduced mtDNA recovery in genome skimming experiments. Similarly, genome skimming is anticipated to be less effective for sponges with high microbial abundances (HMA) than for those with low microbial abundances (LMA), since a considerable proportion of sequenced reads will be derived from symbiotic microorganisms instead of the host sponge. But despite poecilosclerid species being regarded as LMA sponges (*e.g.*, Gloeckner et al., 2014; Thomas et al., 2016), our analyses revealed up to 10-fold variation in exogenous read content among samples.

As metagenomic datasets expand, we will eventually discover how reliable microbial associations are for sponge systematics. Our broad-scale application of genome skimming across familial and ordinal groups found no consistent phylogenetic signal in microbiome composition, which should not be interpreted as a general refutation of microbiome co-evolution in Poecilosclerida. In general, sponges harbour a specific, highly diverse microbial community, comprised mostly by generalist symbionts, with little correlation to environmental communities (Thomas et al., 2016). Our data cannot speak of the potential for microbiomes to exhibit commonality or co-speciation at lower taxa levels, where host evolutionary relationships and geographic isolation often exert a stronger influence on symbiotic associations. However, even studies at smaller evolutionary and geographic scales could not observe co-speciation patterns, despite still finding sponge-specific associations within low abundance bacteria (Reveillaud et al., 2014). Contrary to microbiome composition in HMA sponges, in which environmental filtering and stochastic acquisition play a diminished role (Thomas et al., 2016), in LMA sponges, such as poecilosclerids, the acquisition of functionally relevant bacteria from surrounding waters may be a determinant, as was the case in the *Mycale* association with nitrite-oxidizing bacteria (Reveillaud et al., 2014). Microbiomes in poecilosclerids are seemingly dominated by a few high-abundance species, with high intraspecific variability (Croué et al., 2013; Storey et al., 2020; Waterworth et al., 2021), in direct contrast to the richer and more even microbial communities found in HMA sponges (Erwin et al., 2015; Thomas et al., 2016). Collectively, these constrained features of poecilosclerid microbiomes suggest that microbial communities could be better interpreted as dynamic indicators of habitat or physiology than shared evolutionary history (Gan et al., 2024). Until now, the usefulness of microbiomes for the systematic framework of Poecilosclerida remains to be proved.

## Conclusions

In this study, genome skimming was successfully employed to recover both host sponge and metagenomic sequences from fifteen marine sponges from the order Poecilosclerida. Poriferan-derived sequences constituted the majority of the short reads generated here, facilitating the assembly of four complete mitochondrial genomes and full rDNA clusters for most specimens. The mitogenomes of *Clathria* (*Microciona*) sp., *Desmapsamma anchorata*, *Latrunculia biformis*, and *Neopodospongia tupecomareni* were characterized in detail, revealing several unusual features including an in-frame fusion of the *nd3*::*nd4L* genes and a putative intron within the *trnN(guu)* gene in *D. anchorata*, and a non-canonical UUG start codon for the *cytb* gene in *Clathria* sp. Comparative analysis of poecilosclerid mitochondrial genomes indicated divergence in gene order varied among lineages, with some groups exhibiting relatively conserved structures while others, particularly within Cladorhizidae, Guitarridae, and Mycalidae, appear to possess extensively rearranged, or hyperrearranged mitogenomes. Phylogenetic reconstructions placed *Phlyctaenopora* far from *Mycale* spp., undermining the current Mycalidae framework, and highlighted systematic inconsistencies within and among other families, such as Acarnidae, Crellidae, Coelosphaeridae, Hymedesmiidae, and Microcionidae. This study confirms the suitability of genome skimming for phylogenetic research in these non-model marine invertebrates and underscores the need to develop improved, cost-effective library preparation methods tailored to the unique genomic characteristics of sponges, an approach further extended here to Poecilosclerida, the largest poriferan order.

## Supplemental Information

10.7717/peerj.21781/supp-1 Supplemental Information 1 Supplemental tables and figures (A) Accession numbers of sequences used in this study. (B) Supplementary figures. (C) Features of the mitochondrial genomes assembled in this study.

## Additional Information and Declarations

### Data Availability

The following information was supplied regarding data availability: the code is available at GitHub and Zenodo: - https://github.com/depaulats/Mitogenomes/tree/v.2024.1
- Thiago S. de Paula. (2024). depaulats/Mitogenomes: v.2024.1 (Version v.2024.1) [Computer software]. Zenodo. https://doi.org/10.5281/zenodo.13244412
The data is available at Figshare: de Paula, Thiago (2026). Partial mitochondrial gene sequences generated in the study “Genome skimming of Poecilosclerida (Porifera) reveals conserved and hyperrearranged mitochondrial genomes”. figshare. Dataset. https://doi.org/10.6084/m9.figshare.30742643.v1
The raw sequence data are available at NCBI BioProject: PRJNA1370249, PX693172–PX693175 (mtDNA), PX677315–PX677329 (rDNA), and BK075463–BK075471 (TPA: rDNA).
