# Supplementary material for: Genome skimming of Poecilosclerida (Porifera) reveals conserved and hyperrearranged mitochondrial genomes
Source: PeerJ. 2026 Nov 11;14:e21781. doi: 10.7717/peerj.21781 (PMC13769278; doi:10.7717/peerj.21781)
Supplement: Supplementary file 1 [file peerj-14-21781-s001.pdf]

Genome skimming of Poecilosclerida (Porifera) reveals conserved and hyperrearranged mitochondrial genomes

Thiago Silva de Paula[1], Dora de Moura Barbosa Leite[2], Fabiano Thompson[3], and Eduardo Hajdu[2]

[1]**Departamento de Genética, Universidade do Estado do Rio de Janeiro, Rio de Janeiro, Brazil**
[2]**Museu Nacional, Universidade Federal do Rio de Janeiro, Rio de Janeiro, Brazil**
[3]**Departamento de Biologia Marinha, Universidade Federal do Rio de Janeiro, Rio de Janeiro, Brazil**

Corresponding author:
Thiago S. de Paula[1]

Email address: depaula_ts@hotmail.com, depaula_ts@uerj.br

ABSTRACT

Genome skimming is a cost-effective approach that efficiently recovers multi-copy genomic regions, making it particularly valuable for phylogenetic research and biodiversity assessments. In this study, we applied shallow shotgun metagenome sequencing to 15 poecilosclerid sponges, successfully assembling complete mitochondrial genomes for four species and full rDNA clusters for the majority of samples, despite considerable variation in the retrieval of genomic regions and exogenous, non-poriferan sequences. Phylogenetic reconstructions using both rDNA and mtDNA sequences uncovered extensive polyphyly within several key families, such as Coelosphaeridae and Mycalidade, underscoring significant systematic inconsistencies in generic, familial, and ordinal levels, and emphasizing the need for comprehensive taxonomic revision throughout Poecilosclerida to reflect natural groups. The mitogenomes obtained from *Clathria* (*Microciona*) sp. (18,725 bp), *Desmapsamma anchorata* (19,099 bp), *Latrunculia biformis* (19,630 bp), and *Neopodospongia tupecomareni* (19,983 bp) exhibited conserved architectural features typical of the order, alongside notable variations such as gene overlaps, non-canonical start codons, and an unusual fused *nd3*::*nd4L* gene. Our findings highlight the applicability of genome skimming for phylogenetics and metagenomic insights, though success can vary due to biological and methodological factors. This approach proves to be a cost-effective and powerful tool for advancing sponge systematics, despite challenges in assembling hyperrearranged mitogenomes in certain lineages, paving the way for broader genomic surveys in non-model organisms.

APPENDIX A

Accession numbers of sequences used in this study

STable 1. List of mitochondrial genomes used in this study.

Family	Species	Length (bp)	Accession	Reference
(outgroup)	*Halichondria okadai* (Hok)	20,722	NC_037391	Kim et al., 2017
Acarnidae	*Iophon methanophila* (Ime)	18,718	BK068138	Leite et al., 2024
Cladorhizidae	*Lycopodina hypogea* (Lhy)	31,099	PP657140<br>PP657141<br>PP657142	De Paula et al., 2024
Coelosphaeridae	*Lissodendoryx colombiensis* (Lco)	18,252	OM729635	Lavrov et al., 2023
Crambeidae	*Crambe crambe*	19,532	OZ004599	–
Crellidae	*Crella elegans* (Cel)	18,543	NC_027520	Pett and Lavrov, 2015
**Desmacididae**	***Desmapsamma anchorata* (Dan)**	**19,099**	**PX693173**	***This study***
Hymedesmiidae	*Hymedesmia methanophila* (Hme)	18,657	BK068137	Leite et al., 2024
Hymedesmiidae	*Hymedesmia* sp. (Hsp)	18,716	OM729626	Lavrov et al., 2023
Hymedesmiidae	*Phorbas amaranthus* (Pam)	18,568	OM729646	Lavrov et al., 2023
Hymedesmiidae	*Phorbas areolatus* (Par)	18,601	MK079948	Plese et al., 2021
Hymedesmiidae	*Phorbas tenacior* (Pte)	17,070	MK079949	Plese et al., 2021
Iotrochotidae	*Iotrochota birotulata* (Ibi)	19,112	NC_010207	Wang and Lavrov, 2008
**Latrunculiidae**	***Latrunculia biformis* (Lap)**	**19,630**	**PX693174**	***This study***
**Microcionidae**	***Clathria* (*Microciona*) sp. (CMsp)**	**18,725**	**PX693172**	***This study***
Microcionidae	*Clathria curacaoensis* (Ccu)	18,845	OM729636	Lavrov et al., 2023
Podospongiidae	*Diacarnus erythraeanus* (Der)	19,339	OZ024687	–
Podospongiidae	*Negombata magnifica* (Nma)	20,088	NC_010171	Belinky et al., 2008
**Podospongiidae**	***Neopodospongia tupecomareni* (Ntu)**	**19,983**	**PX693175**	***This study***
Tedaniidae	*Tedania ignis* (Tig)	19,295	OM729644	Lavrov et al., 2023
–	Poecilosclerida sp. (Psp)	18,587	OM729630	Lavrov et al., 2023
Desmacellidae[D]	*Desmacella informis* (Din)	12,900*	OM729615	Lavrov et al., 2023
Hamacanthidae[M]	*Hamacantha johnsoni* (Hjo)	11,135*	OM729619	Lavrov et al., 2023

* Only partial sequences available.
All families from the Order Poecilosclerida but two currently allocated into orders Desmacellida ([D]) e Merliida ([M]) are indicated.
Sequences generated in this study are in bold.
Partial mtDNA sequences generated in this study available at https://doi.org/10.6084/m9.figshare.30742643.

STable 2. List of 18S and 28S rDNA sequences used in this work.

Family	Species	Voucher	Acession	
			18S	28S
(outgroup)	*Halichondria okadai*	-	AB511881	
Acarnidae	*Iophon metanophila*	UPSZTY 167253	BK068394	
**Cladorhizidae**	***Asbestopluma pennatula***	**ZMBN 148461**	**PX677315**	
**Cladorhizidae**	***Chondrocladia grandis***	**ZMBN 148469**	**PX677316**	
**Cladorhizidae**	***Cladorhiza gelida***	**ZMBN 148466**	**PX677317**	
Cladorhizidae	*Lycopodina hypogea*	MNRJ 22102	PP658212	
Coelosphaeridae	*Forcepia* sp.	NCI034	KC902407	KC869627
Coelosphaeridae	*Inflatella* sp.	NCI385	KC902061	KC869468
Coelosphaeridae	*Lissodendoryx arenaria*	NCI321	KC901932	KC869561
Coelosphaeridae	*Lissodendoryx fibrosa*	NCI401	KC901939	KC869529
Coelosphaeridae	*Lissodendoryx sigmata*	NCI429	KC902121	KC869509
**Coelosphaeridae**	***Lissodendoryx isodictyalis***	**MNRJ 10474**	**PX677324**	
Crambeidae	*Crambe crambe*	UCMPWC933	EF654524	AY561883
Crambeidae	*Monanchora arbuscula*	SI06x202	KC902187	KC869447
Crellidae	*Crella incrustans*	NCI025	KC902141	KC869608
**Desmacididae**	***Desmapsamma anchorata***	**MNRJ 22097**	**PX677319**	
Esperiopsidae	*Amphilectus fucorum*	Mc5093	KC902221	HQ379226+<br>HQ379294+<br>HQ379362
**Esperiopsidae**	***Ulosa digitata***	**ZMAPOR 14109**	**PX677329**	
Guitarridae	*Guitarra fimbriata*	NCI405	KC902210	KC869537
**Guitarridae**	***Guitarra sepia***	**MNRJ 22099**	**PX677321**	
Hymedesmiidae	*Hymedesmia metanophila*	UPSZTY 167252	BK068393	
Iotrochotidae	*Iotrochota birotulata**	P01	KC902139	KC869556
Isodictyidae	*Isodictya frondosa*	NCI381	KC902023	KC869563
**Latrunculiidae**	***Latrunculia biformis***	**MNRJ 11166**	**PX677323**	
Latrunculiidae	*Latrunculia lunaviridis*	NCI417	KC902327	KC869489
Latrunculiidae	*Tsitsikamma pedunculata*	NCI384	KC902279	KC869512
Microcionidae	*Antho* cf. *prima*	NCI322	KC902142	KC869629
**Microcionidae**	***Clathria* (*Microciona*) sp.**	**MNRJ 20519**	**PX677318**	
**Microcionidae**	***Clathria prolifera***	**SRR22301254**	**BK075463**	
Microcionidae	*Clathria reinwardti*	NCI453	KY947253**<br>∼KX894462**	KC869449
Mycalidae	*Anomomycale titubans*	Mc7765	KC902230	HQ379228+<br>HQ379297+<br>HQ379365
**Mycalidae**	***Mycale* (*Grapelia*) sp.**	**MNRJ 22646**	**PX677325**	
**Mycalidae**	***Mycale* (*Naviculina*) *diversisigmata***	**MNRJ 22115**	**PX677326**	
**Mycalidae**	***Mycale acerata***	**SRR11291276**	**BK075464**	
**Mycalidae**	***Mycale cecilia*, green morph**	**SRR13082362**	**BK075465**	
**Mycalidae**	***Mycale cecilia*, red morph**	**SRR13082360**	**BK075466**	
Mycalidae	*Mycale fibrexilis*	-	AF100946	AY026376
**Mycalidae**	***Mycale grandis***	**SRR3334579**	**BK075467**	
**Mycalidae**	***Mycale laevis***	**SRR11294007**	**BK075468**	
Mycalidae	*Mycale macilenta*	NCI346	KC901929	KC869541
Mycalidae	*Mycale mirabilis*	NCI445	KC902146	KC869613
**Mycalidae**	***Mycale phyllophila***	**SRR2394941**	**BK075469**	
**Mycalidae**	***Mycale tridens***	**SRR11303650**	**BK075470**	
**Mycalidae**	***Phlyctaenopora* (*Barbozia*) sp.**	**MNRJ 17428**	**PX677328**	
Myxillidae	*Myxilla* cf. *rosacea*	Mc4681	KC901935	HQ379231+<br>HQ379403+<br>HQ379403+<br>HQ379403
Ophlitaspongiinae	*Echinochalina* cf. *laboutei*	NCI468	KC901908	KC869603
Ophlitaspongiinae	*Echinoclathria dichotoma*	NCI444	KC901933	KC869501
**Podospongiidae**	***Diacarnus megaspinorhabdosa***	**ZMAPOR 13278**	**PX677320**	
Podospongiidae	*Negombata magnifica*	-	FR819687	OM406234
**Podospongiidae**	***Neopodospongia tupecomareni***	**MNRJ 10323b**	**PX677327**	
**Tedaniidae**	***Tedania ignis***	**ERR2756782**	**BK075471**	
Tedaniidae	*Tedania strongylostyla*	NCI397	KC901911	KC869515
Desmacellidae[D]	*Desmacella* cf. *annexa*	Mc4240a	KC902284	HQ379225+<br>HQ379293+<br>HQ379361
**Hamacanthidae[M]**	***Hamacantha falcula***	**ZMAPOR 19419**	**PX677322**	

*Erroneously identified as *Mycale laevis* in the database (T.S de Paula, pers. obs.).
**Sequences not from the same voucher.
A plus sign (+) indicates that data in the matrix was a composition of partial deposited sequences.
All families from the Order Poecilosclerida but two allocated into Desmacellida ([D]) e Merliida ([M]).
Sequences generated in this study are in bold.

STable 3. Taxonomic classification of reads in Poecilosclerida genome skimming.

Taxon	Ape	Cge	Cgr	CTsp	Dan	Dme	Gse	Hfa	Lbi	Lsp	MGsp	MNsp	Ntu	PGsp	Udi
Acidobacteriota	0.080	-	-	-	0.013	3.113	0.056	0.063	0.041	0.040	0.040	0.011	0.017	3.402	-
Actinomycetota	2.513	0.246	19.907	4.348	3.472	84.557	0.311	5.942	4.305	16.864	1.774	3.197	13.307	97.326	2.599
Apicomplexa	5.226	0.893	1.935	5.479	0.877	0.055	1.136	1.489	4.670	0.315	1.814	1.936	0.355	1.565	5.949
Aquificota	-	-	-	-	-	-	0.007	-	-	-	-	0.011	-	-	-
Archaea	0.013	0.665	0.102	-	0.013	-	-	2.076	0.041	-	0.020	-	0.818	0.188	-
Armatimonadota	-	-	-	-	-	0.018	-	-	-	-	-	-	-	0.021	-
Ascomycota	4.304	2.169	9.114	9.827	1.867	0.183	1.683	2.963	8.041	0.385	1.411	1.759	0.620	1.294	4.791
Bacillariophyta	0.040	-	0.025	0.119	0.050	-	0.045	0.063	0.569	0.020	0.020	0.044	0.107	0.125	0.344
Bacillota	2.793	1.704	2.800	0.953	5.590	3.590	0.435	2.789	1.624	0.409	0.766	0.597	3.447	4.049	4.196
Bacteria	99.238	66.394	142.406	27.455	143.054	423.061	10.643	194.550	90.156	139.130	31.330	38.490	223.908	374.423	95.779
Bacteroidota	12.323	15.020	17.947	1.012	16.556	1.465	2.309	10.441	22.823	0.839	0.484	2.124	32.508	1.565	43.459
Balneolota	-	-	-	-	0.013	-	-	-	0.041	-	-	-	0.008	-	-
Basidiomycota	0.949	0.574	1.146	0.060	0.063	0.092	0.139	0.174	0.162	-	0.101	0.044	0.182	0.042	0.094
Bdellovibrionota	-	0.018	-	-	0.013	0.018	0.004	-	-	0.005	-	-	0.017	0.104	-
Calditrichota	0.013	-	-	-	-	0.055	-	0.016	-	-	-	-	-	-	-
Campylobacterota	0.495	0.100	0.127	-	0.363	0.110	0.127	0.269	0.244	0.040	0.081	0.022	0.306	0.021	0.344
Candidatus Bipolaricaulota	-	-	-	-	-	0.018	-	-	-	-	-	-	-	-	-
Candidatus Cloacimonadota	-	-	-	-	-	-	-	0.016	-	-	-	-	-	0.042	-
Candidatus Saccharibacteria	-	-	-	-	-	-	-	0.016	-	-	-	-	-	0.063	-
Candidatus Thermoplasmatota	-	-	0.025	-	-	-	0.007	-	-	-	-	-	-	0.021	-
Cercozoa	-	-	-	-	-	-	-	0.016	-	-	-	-	-	-	-
Chlamydiota	-	-	-	-	-	0.018	0.004	0.032	-	-	-	0.011	0.008	-	0.063
Chlorobiota	0.027	0.036	0.076	-	-	0.110	-	0.032	-	0.065	-	-	0.025	0.042	-
Chloroflexota	0.334	0.100	0.153	-	0.025	2.436	-	0.032	-	0.020	0.020	-	0.033	2.338	-
Chordata	368.952	295.706	348.760	549.163	152.040	30.512	856.523	219.805	534.600	22.617	814.016	792.846	24.036	132.099	457.887
Cossaviricota	-	-	-	-	-	-	-	0.032	-	-	-	-	-	-	-
Cyanobacteriota	3.061	3.472	1.273	19.534	120.670	45.420	9.508	7.066	0.690	9.938	5.726	26.121	36.806	1.294	17.440
Deferribacterota	0.013	0.009	-	-	0.025	0.018	-	-	-	-	-	-	-	0.021	-
Deinococcota	-	0.009	-	-	0.013	0.458	0.004	0.127	-	0.055	0.020	0.033	0.223	0.647	-
Elusimicrobiota	-	-	-	-	-	0.018	-	-	-	-	-	-	-	-	-
Euglenozoa	0.067	-	0.127	0.179	0.038	-	0.075	0.016	0.081	0.005	0.020	0.022	0.033	-	0.031
Eukaryota	22.761	11.893	15.631	-	-	-	70.327	-	-	-	17.943	0.011	-	27.071	-
Euryarchaeota	0.348	0.009	0.178	0.179	0.063	0.733	0.007	0.586	0.041	0.020	0.101	0.321	0.041	0.835	0.125
Evosea	0.027	-	-	-	0.013	-	0.045	0.016	-	-	0.020	0.011	0.017	-	0.031
Fusobacteriota	0.013	0.018	0.051	0.060	0.013	0.018	0.011	0.032	-	0.015	0.040	-	0.017	-	0.031
Gemmatimonadota	-	0.009	0.102	-	-	0.238	-	0.032	0.081	-	-	0.011	-	0.250	-
Kiritimatiellota	-	-	0.025	-	-	0.220	-	-	-	-	-	-	-	0.042	-
Microsporidia	-	-	-	-	-	-	-	-	-	-	-	-	-	0.021	0.063
Mycoplasmatota	0.013	0.055	0.076	-	0.050	0.018	0.007	0.158	0.081	-	0.101	0.022	-	0.104	0.031
Myxococcota	0.027	0.009	-	0.238	0.150	3.333	-	0.063	0.041	0.080	-	0.221	0.479	1.920	-
Nitrososphaerota	36.608	416.017	75.938	0.774	0.050	0.110	0.750	116.280	15.513	0.125	7.056	0.111	57.717	9.205	5.824
Nitrospirota	-	-	0.051	0.060	-	0.842	-	-	-	-	0.081	-	1.446	0.793	-
Nucleocytoviricota	0.027	-	0.051	-	0.401	0.055	0.101	1.378	1.056	-	0.020	0.022	0.149	-	3.194
Peploviricota	-	-	-	0.060	-	-	0.101	-	-	-	0.020	-	-	-	-
Planctomycetota	0.120	0.146	0.051	0.298	0.238	5.128	0.022	2.662	0.893	0.330	0.121	0.575	0.554	3.507	0.971
Pseudomonadota	373.349	153.913	320.987	205.289	503.158	358.430	18.967	372.257	159.154	791.986	85.200	72.035	568.516	276.200	200.607
Rhodothermota	-	-	-	-	-	0.513	-	0.048	-	0.020	-	-	-	0.313	-
Spirochaetota	0.040	0.082	0.178	-	0.088	0.220	0.004	0.032	0.122	0.005	0.040	0.111	0.041	0.209	-
Synergistota	0.013	-	-	-	0.113	0.037	-	-	0.041	0.005	-	-	-	0.063	-
Thermodesulfobacteriota	0.601	0.191	0.229	0.119	3.234	1.044	0.056	0.174	0.731	0.325	-	0.144	0.471	1.252	0.282
Thermodesulfobiota	-	-	-	-	-	-	-	-	-	-	-	-	0.017	0.021	-
Thermomicrobiota	-	-	-	-	-	0.110	-	-	-	-	-	-	-	0.104	-
Thermoproteota	-	0.018	0.204	-	-	-	0.007	0.032	0.041	-	-	-	-	-	-
Thermosulfidibacterota	-	-	-	-	-	-	-	-	-	-	-	0.022	-	-	-
Thermotogota	-	0.009	-	-	0.013	0.110	-	-	-	-	-	-	-	0.042	-
Uroviricota	0.053	-	0.025	3.037	9.438	1.190	0.210	0.444	0.081	1.288	2.157	1.339	0.975	0.584	1.566
Verrucomicrobiota	0.027	0.009	0.051	0.060	0.201	0.128	0.026	0.190	0.041	0.055	0.020	0.022	0.157	0.417	0.157
Viruses	0.040	0.027	0.025	71.586	12.696	1.099	0.011	14.592	75.333	2.796	-	25.745	2.785	-	42.301
root	65.491	30.476	40.222	100.113	25.330	31.098	26.331	43.001	78.663	12.205	29.435	32.007	29.854	56.354	111.842
Total reads (count)	74820	109724	39282	16791	79788	54602	266838	63115	24624	200251	49601	90387	120987	47911	31938
Total reads (‰)	23.174	40.526	16.024	11.801	24.975	40.901	80.020	32.864	8.477	64.078	18.771	24.435	51.005	22.616	17.657

Species abbreviattions as: Ape, *Asbestopluma pennatula*; Cge, *Cladorhiza gelida*; Cgr, *Chondrocladia grandis*; CMsp, *Clathria* (*Microciona*) sp.; Dan, *Desmapsamma anchorata*; Dme, *Diacarnus megaspinorhabdosa*; Gse, *Guitarra sepia*; Hfa, *Hamacantha falcula*; Lap, *Latrunculia biformis*; Lis, *Lissodendoryx isodictyalis* sp.; Mdi, *Mycale* (*Naviculina*) *diversisigmata*; MGsp, *Mycale* (*Grapelia*) sp.; Ntu, *Neopodospongia tupecomareni*; PBsp, *Phlyctaenopora* (*Barbozia*) sp.; Udi, *Ulosa digitata*.

As per the classification results of Kraken2 with PlusPF database v.2022-06-07. Permillage (‰) of classified reads among taxa in proportion to total classified reads. Permillage (‰) of total classified reads in proportion to filtered reads in the dataset.

REFERENCES

Belinky, F., Rot, C., Ilan, M., and Huchon, D. (2008). The complete mitochondrial genome of the demosponge *Negombata magnifica* (Poecilosclerida). *Molecular Phylogenetics and Evolution*, 47(3):1238–1243.

De Paula, T. S., Leite, D. D. M. B., Lobo-Hajdu, G., Vacelet, J., Thompson, F., and Hajdu, E. (2024). The complete mitochondrial DNA of the carnivorous sponge *Lycopodina hypogea* is putatively complemented by microDNAs. *PeerJ*, 12:e18255.

Kim, H., Kim, H. J., Jung, Y. H., Yu, C., An, Y. R., Han, D., and Kang, D. W. (2017). The complete mitochondrial genome of sponge *Halichondria okadai* (Demospongiae, Suberitida, Halichondriidae) from Korea water. *Mitochondrial DNA Part B*, 2(2):873–874.

Lavrov, D. V., Diaz, M. C., Maldonado, M., Morrow, C. C., Perez, T., Pomponi, S. A., and Thacker, R. W. (2023). Phylomitogenomics bolsters the high-level classification of Demospongiae (phylum Porifera). *PLOS ONE*, 18(12):e0287281.

Leite, D. D. M. B., De Paula, T. S., and Hajdu, E. (2024). The complete mitochondrial genome of the deep-sea methanotrophic sponges *Hymedesmia methanophila* and *Iophon methanophila*: leveraging 'waste' in metagenomic data. *Journal of Genetics*, 103(2):34.

Pett, W. and Lavrov, D. V. (2015). Cytonuclear Interactions in the Evolution of Animal Mitochondrial tRNA Metabolism. *Genome Biology and Evolution*, 7(8):2089–2101.

Plese, B., Kenny, N. J., Rossi, M. E., Cárdenas, P., Schuster, A., Taboada, S., Koutsouveli, V., and Riesgo, A. (2021). Mitochondrial evolution in the Demospongiae (Porifera): Phylogeny, divergence time, and genome biology. *Molecular Phylogenetics and Evolution*, 155:107011.

Wang, X. and Lavrov, D. V. (2008). Seventeen New Complete mtDNA Sequences Reveal Extensive Mitochondrial Genome Evolution within the Demospongiae. *PLoS ONE*, 3(7):e2723.

APPENDIX B

Supplementary figures

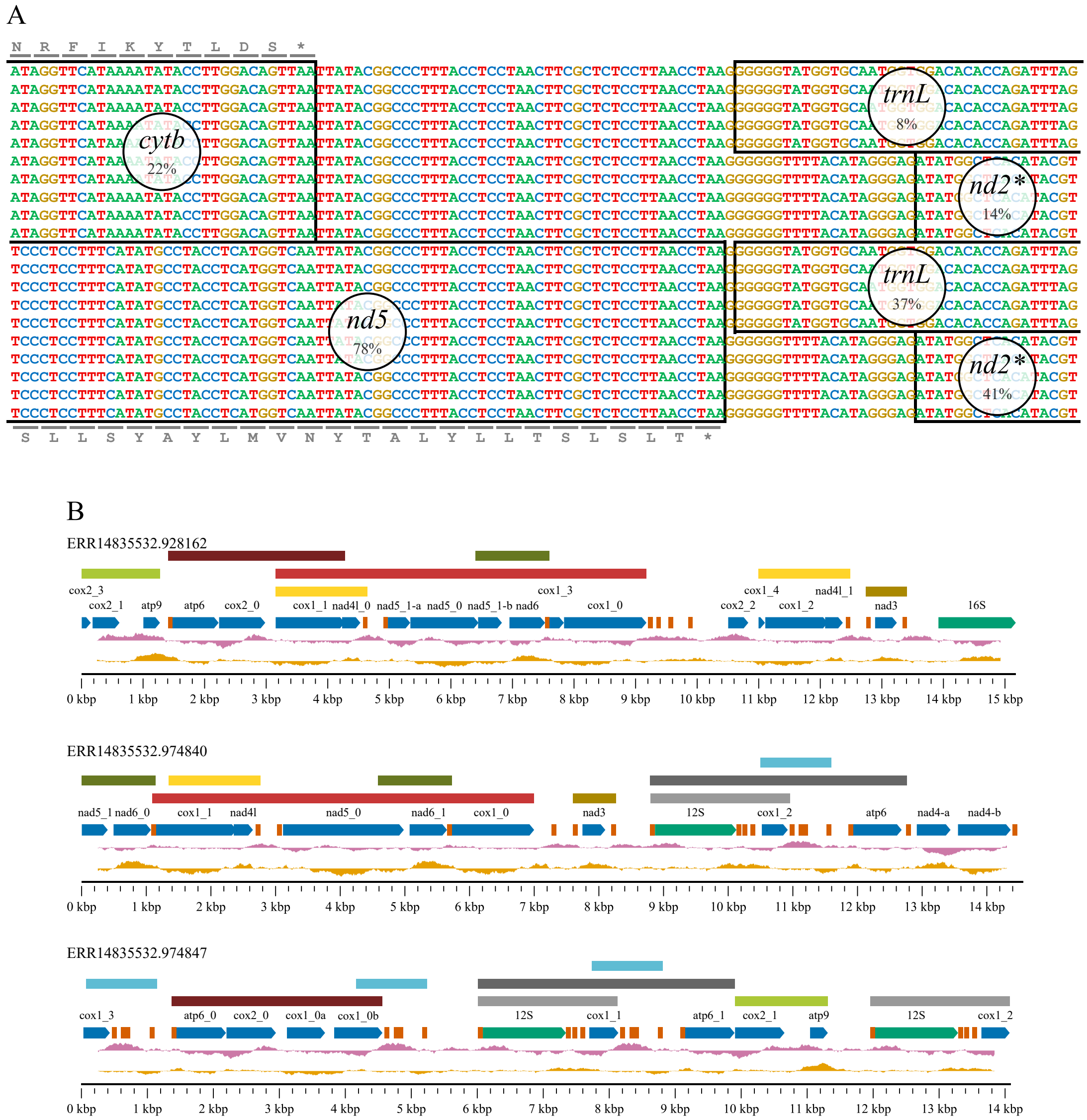

SFigure 1. Putative hyperrearranged mtDNA sequences in Poecilosclerida. A. Illumina short reads in the metagenome of *Guitarra sepia*. Alignment of filtered reads mapped against a mtDNA reference, spanning the conserved motif 'AAT TAT ACG GCC CTT TAC CTC CTA ACT TCG CTC TCC TTA ACC TAA GGG GGG T'. Flanking sequence variants comprising particular genes and their relative abundance among reads. Protein-coding genes translated according to the sponge/mold mitochondrial genetic code. The *nd2* gene is annotated by its alt-ORF (*), which likely start site is shifted 19 codons downstream. B. PacBio HiFi long reads in *Mycale lingua* (ERR14835532). The three longest reads with positive hits for Poecilosclerida mtDNA genes. Annotations were conducted as before with MITOS2 and regions with high similarity among reads are highlighted with colored bars.

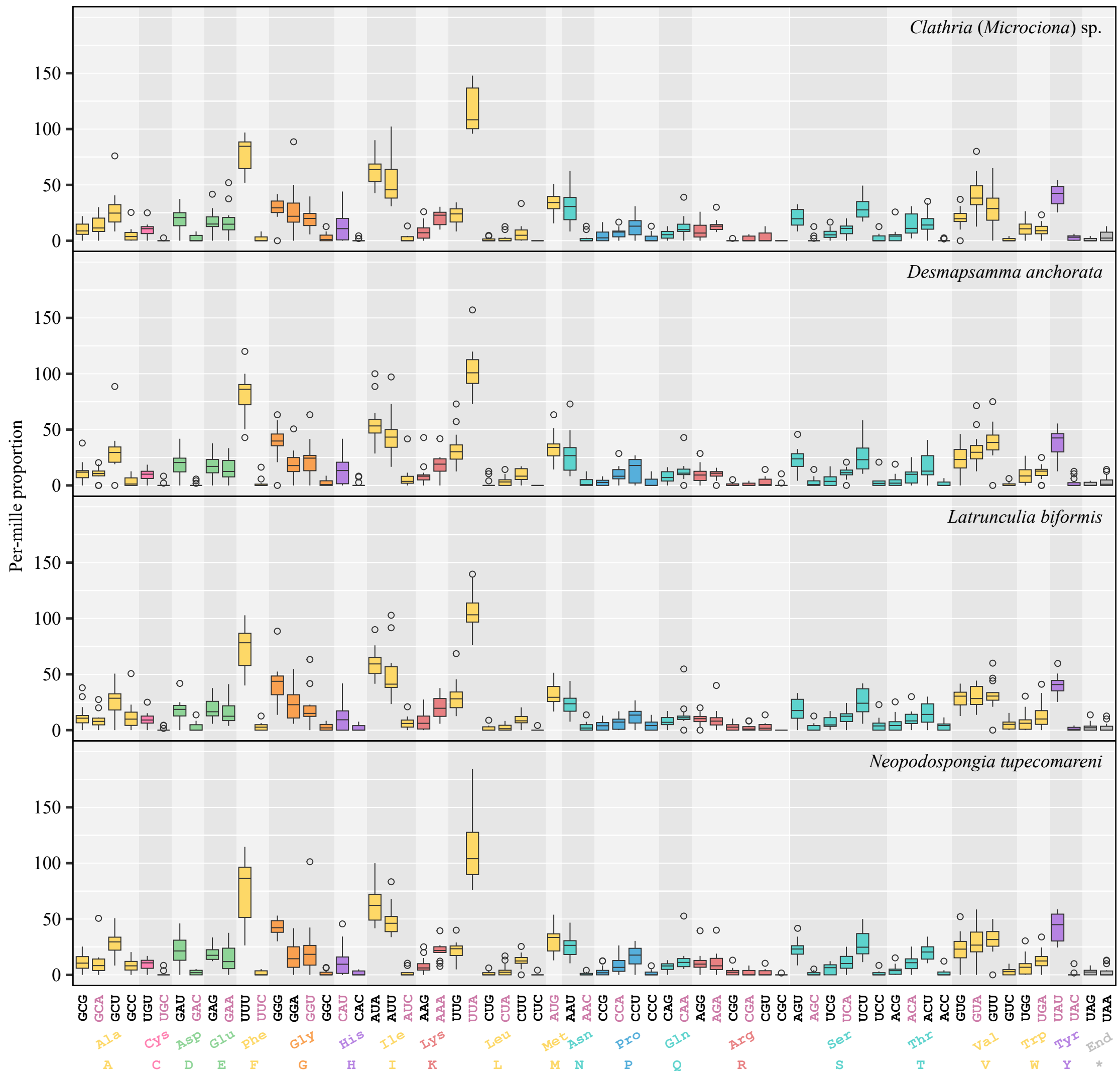

SFigure 2. Codon usage in mitochondrial genomes. Amino acids biochemical properties presented in colors. Codons corresponding to mtDNA-encoded tRNAs are highlighted.

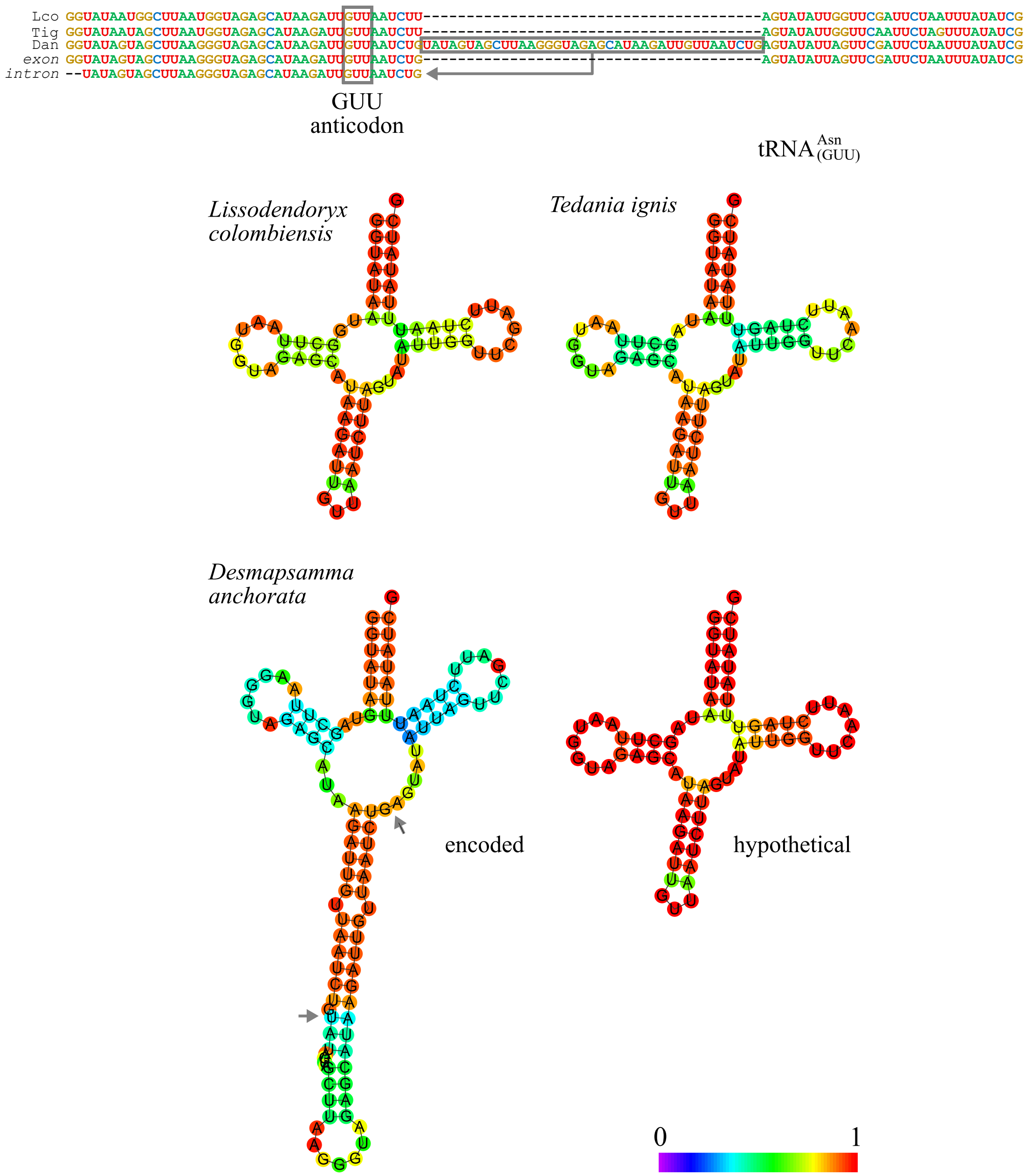

SFigure 3. Comparison among $tRNA^{Asn}_{GUU}$ in the mitogenome of *Desmapsamma anchorata* and closer relatives. The gene enconded in *D. anchorata* presents a 39 nt intron comprising a duplication from upstream. Secondary structure of the sequence currently encoded in *D. anchorata* mitogenome and the hypothetical version in relation to the others. Arrows show the intron start and end positions. For comparative sequences, see Appendix A.

APPENDIX C

Features of the mitochondrial genomes assembled in this study

STable 4. Features of the mitochondrial genome of *Clathria* (*Microciona*) sp..

Gene	Position		Length	Amino	Start/Stop	Intergenic	Strand
	Start	End	(bp)	acid	codon	region (bp)	
rnl	1	2,493	2,493	–	–	7	H
trnI(cau)	2,530	2,601	72	–	–	36	H
trnY(gua)	2,623	2,690	68	–	–	21	H
trnK(uuu)	2,691	2,762	72	–	–	0	H
atp8	2,764	2,994	231	76	AUG/UAA	1	H
atp6	2,984	3,718	735	244	AUG/UAG	-11	H
missing[a]	3,285	3,319	35	11 2/3	–	–	H
trnR(ucu)	3,727	3,798	72	–	–	8	H
cox3	3,827	4,615	789	262	AUG/UAA	28	H
missing[a]	3,850	3,953	104	34 2/3	–	–	H
trnQ(uug)	4,629	4,703	75	–	–	13	H
trnW(uca)	4,710	4,780	71	–	–	6	H
trnL(uag)	4,896	4,969	74	–	–	115	H
trnN(guu)	4,973	5,043	71	–	–	3	H
cytb	5,045	6,202	1,158	385	UUG[b]/UAA	1	H
trnT(ugu)	6,213	6,285	73	–	–	10	H
atp9	6,321	6,557	237	78	AUG/UAA	35	H
trnS(gcu)	6,572	6,658	87	–	–	14	H
nd4	6,858	8,306	1,449	482	GUG/UAG	199	H
trnH(gug)	8,322	8,393	72	–	–	15	H
trnR(ucg)	8,495	8,566	72	–	–	101	H
trnE(uuc)	8,575	8,645	71	–	–	8	H
nd6	8,643	9,179	537	178	GUG/UAA	-3	H
nd3	9,251	9,610	360	119	AUG/UAA	71	H
nd4L	9,629	9,928	300	99	AUG/UAA	18	H
cox1	10,008	11,558	1,551	516	GUG/UAG	79	H
trnS(uga)	11,549	11,627	79	–	–	-10	H
trnC(gca)	11,637	11,710	74	–	–	9	H
nd1	11,732	12,703	972	323	AUG/UAG	21	H
trnL(uaa)	12,707	12,780	74	–	–	3	H
trnI(gau)	12,786	12,858	73	–	–	5	H
trnM(cau)	12,881	12,950	70	–	–	22	H
nd2	12,957	14,336	1,380	459	GUG/UAA	6	H
nd5	14,364	16,241	1,878	625	AUG/UAA	27	H
trnA(ugc)	16,246	16,318	73	–	–	4	H
cox2	16,368	17,087	720	239	AUG/UAG	49	H
trnF(gaa)	17,248	17,320	73	–	–	160	H
rns	17,322	18,558	1,237	–	–	1	H
trnG(ucc)	18,559	18,630	72	–	–	0	H
trnV(uac)	18,648	18,718	71	–	–	17	H

[a] Missing data (N) according to regions from the *Negombata magnifica* mitogenome (NC_010171).
[b] Non-canonical start codon.

STable 5. Composition and skewness in the mitochondrial genome of *Clathria* (*Microciona*) sp..

Gene/Region	G	A	T	C	GC%	AT-skew	GC-skew	Length (bp)
atp6[a]	18.0	27.1	41.9	8.3	26.3	-0.21499	0.36788	735
atp8	18.2	34.6	38.1	9.1	27.3	-0.04762	0.33333	231
atp9	24.1	28.3	36.3	11.4	35.4	-0.12418	0.35714	237
cox1	21.9	27.0	39.4	11.8	33.7	-0.18756	0.29885	1,551
cox2	23.6	31.0	35.4	10.0	33.6	-0.06695	0.40496	720
cox3[a]	20.2	22.9	33.8	9.9	30.0	-0.19196	0.34177	789
cytb	20.8	28.9	41.8	8.5	29.3	-0.18193	0.42183	1,158
nd1	21.7	27.2	42.9	8.2	29.9	-0.22467	0.45017	972
nd2	20.8	29.0	42.4	7.8	28.6	-0.18782	0.45316	1,380
nd3	21.7	26.9	43.3	8.1	29.7	-0.23320	0.45794	360
nd4	20.6	26.6	42.7	10.1	30.7	-0.23108	0.33933	1,449
nd4L	19.3	34.7	40.3	5.7	25.0	-0.07556	0.54667	300
nd5	20.2	28.3	42.4	9.1	29.3	-0.19880	0.37818	1,878
nd6	18.4	29.8	46.4	5.4	23.8	-0.21760	0.54688	537
rnl	22.5	36.7	29.6	11.2	33.7	0.10708	0.33810	2,493
rns	24.3	35.8	30.5	9.4	33.7	0.08049	0.44365	1,237
tRNAs[b]	24.8	29.3	33.4	12.5	37.3	-0.06640	0.33000	1,609
IGRs[c]	20.4	42.5	32.6	4.5	24.9	0.13199	0.64029	1,119
mtDNA[d]	21.5	30.7	37.6	9.4	31.0	-0.10220	0.39072	18,725

[a] Missing data (N, %): *atp6* = 4.8; *cox3* = 13.2.
[b] Totaling 22 tRNA genes.
[c] Encompassing 34 intergenic regions (Q_1 = 6, Q_2 = 15, Q_3 = 33, Q_4 = 199).
[d] Discounting 21 bp from overlapping genes.

STable 6. Features of the mitochondrial genome of *Desmapsamma anchorata*.

Gene	Position		Length	Amino	Start/Stop	Intergenic	Strand
	Start	End	(bp)	acid	codon	region (bp)	
rnl	1	2,544	2,544	–	–	6	H
trnI(cau)	2,555	2,626	72	–	–	10	H
trnY(gua)	2,631	2,702	72	–	–	4	H
trnK(uuu)	2,729	2,801	73	–	–	26	H
atp8	2,803	3,012	210	69	GUG/UAA	1	H
atp6	3,014	3,748	735	244	AUG/UAG	1	H
trnR(ucu)	3,770	3,841	72	–	–	21	H
cox3	3,864	4,652	789	262	AUG/UAG	22	H
trnQ(uug)	4,670	4,744	75	–	–	17	H
trnW(uca)	4,765	4,835	71	–	–	20	H
trnL(uag)	4,856	4,929	74	–	–	20	H
trnN(guu)	4,934	5,043	110	–	–	4	H
intron 1	4,975	5,013	39	–	–	–	H
cytb	5,045	6,199	1,155	384	AUG/UAA	36	H
trnT(ugu)	6,221	6,292	72	–	–	21	H
atp9	6,334	6,570	237	78	AUG/UAA	41	H
trnS(gcu)	6,590	6,676	87	–	–	19	H
trnR(ucg)	6,724	6,797	74	–	–	47	H
trnP(ugg)	6,970	7,042	73	–	–	172	H
nd4	7,067	8,518	1,452	483	AUG/UAA	24	H
trnH(gug)	8,759	8,830	72	–	–	240	H
trnE(uuc)	8,841	8,911	71	–	–	10	H
nd6	8,909	9,460	552	184	GUA/UAA	-3	H
trnD(guc)	9,482	9,552	71	–	–	21	H
nd3::nd4L[a]	9,580	10,227	648	215	AUG/UAA	27	H
exon *nd3*	9,580	9,936	357	119	–	–	H
exon *nd4L*	9,937	10,227	291	96	–	–	H
cox1	10,481	12,040	1,560	519	AUG/UAG	253	H
trnS(uga)	12,031	12,108	78	–	–	-10	H
trnC(gca)	12,122	12,193	72	–	–	13	H
nd1	12,203	13,174	972	323	AUG/UAG	9	H
trnL(uaa)	13,178	13,251	74	–	–	3	H
trnI(gau)	13,283	13,353	71	–	–	31	H
trnM(cau)	13,456	13,525	70	–	–	102	H
nd2	13,538	14,914	1,377	458	AUG/UAA	12	H
nd5	14,926	16,818	1,893	630	AUG/UAG	11	H
trnA(ugc)	16,827	16,899	73	–	–	8	H
cox2	16,947	17,663	717	238	GUG/UAG	47	H
trnF(gaa)	17,654	17,726	73	–	–	-10	H
rns	17,729	18,933	1,205	–	–	2	H
trnG(ucc)	18,934	19,005	72	–	–	0	H
trnV(uac)	19,022	19,093	72	–	–	16	H

[a] Exons in gene fusion according to amino acids sequence alignments.

STable 7. Composition and skewness in the mitochondrial genome of *Desmapsamma anchorata*.

Gene/Region	G	A	T	C	GC%	AT-skew	GC-skew	Length (bp)
atp6	20.0	25.0	44.0	11.0	31.0	-0.27416	0.28947	735
atp8	19.1	32.4	40.5	8.1	27.2	-0.11111	0.40351	210
atp9	27.0	26.2	35.0	11.8	38.8	-0.14483	0.39130	237
cox1	22.3	25.6	40.1	11.9	34.2	-0.22027	0.30337	1,560
cox2	22.9	30.3	36.3	10.6	33.5	-0.09015	0.36667	717
cox3	24.6	25.4	38.8	11.3	35.9	-0.20949	0.37102	789
cytb	21.1	27.1	42.3	9.5	30.7	-0.21848	0.37853	1,155
nd1	22.7	26.3	41.3	9.7	32.4	-0.22070	0.40317	972
nd2	22.8	26.8	41.5	8.9	31.7	-0.21573	0.44037	1,377
nd3[a]	22.4	26.6	44.0	7.0	29.4	-0.24603	0.52381	357
nd4	22.5	24.9	43.5	9.1	31.6	-0.27291	0.42484	1,452
nd4L[a]	21.0	27.8	41.6	9.6	30.6	-0.19802	0.37079	291
nd5	20.8	27.7	42.3	9.2	30.0	-0.20906	0.38732	1,893
nd6	23.0	23.2	47.5	6.3	29.3	-0.34359	0.56790	552
rnl	24.5	35.7	27.9	11.9	36.4	0.12307	0.34628	2,544
rns	25.9	34.9	29.2	10.0	35.9	0.08808	0.44111	1,205
tRNAs[b]	24.5	28.5	33.8	13.2	37.7	-0.08509	0.29909	1,755
Introns	25.6	33.3	33.3	7.7	33.3	0.00000	0.53846	39
IGRs[c]	24.1	34.6	37.3	4.0	28.1	-0.03727	0.71662	1,306
mtDNA[d]	23.1	29.0	38.0	10.0	33.0	-0.13394	0.39556	19,099

[a] Corresponding exonic regions within the *nd3::nd4L* gene fusion.
[b] Totaling 24 tRNA genes.
[c] Encompassing 36 intergenic regions (Q_1 = 9, Q_2 = 20, Q_3 = 26, Q_4 = 253).
[d] Discounting 20 bp from overlapping genes.

STable 8. Features of the mitochondrial genome of *Latrunculia biformis*.

Gene	Position		Length	Amino	Start/Stop	Intergenic	Strand
	Start	End	(bp)	acid	codon	region (bp)	
rnl	1	2,484	2,484	–	–	44	H
trnI(cau)	2,499	2,570	72	–	–	14	H
trnY(gua)	2,575	2,646	72	–	–	4	H
trnK(uuu)	2,669	2,741	73	–	–	22	H
atp8	2,742	2,960	219	72	AUG/UAG	0	H
atp6	2,950	3,684	735	244	AUG/UAA	-11	H
trnH(gug)	3,722	3,793	72	–	–	37	H
cox3	4,196	4,984	789	262	AUG/UAG	402	H
trnQ(uug)	5,003	5,077	75	–	–	18	H
trnW(uca)	5,134	5,204	71	–	–	56	H
trnL(uag)	5,245	5,317	73	–	–	40	H
trnN(guu)	5,322	5,392	71	–	–	4	H
cytb	5,394	6,548	1,155	384	GUG/UAG	1	H
trnR(ucu)	6,580	6,652	73	–	–	31	H
atp9	6,692	6,928	237	78	GUG/UAA	39	H
trnS(gcu)	6,948	7,034	87	–	–	19	H
trnP(ugg)	7,044	7,116	73	–	–	9	H
nd4	7,146	8,597	1,452	483	AUG/UAG	29	H
trnE(uuc)	8,638	8,709	72	–	–	40	H
nd6	8,707	9,258	552	183	GUG/UAA	-3	H
trnD(guc)	9,291	9,361	71	–	–	32	H
nd3	9,384	9,743	360	119	AUG/UAG	22	H
nd4L	9,755	10,054	300	99	AUG/UAA	11	H
cox1	10,596	12,152	1,557	518	AUG/UAG	541	H
trnS(uga)	12,143	12,221	79	–	–	-10	H
trnC(gca)	12,240	12,311	72	–	–	18	H
nd1	12,331	13,302	972	323	AUG/UAG	19	H
trnL(uaa)	13,349	13,422	74	–	–	46	H
trnT(ugu)	13,426	13,497	72	–	–	3	H
trnR(ucg)	13,584	13,657	74	–	–	86	H
trnI(gau)	14,037	14,110	74	–	–	379	H
trnM(cau)	14,132	14,202	71	–	–	21	H
nd2	14,215	15,567	1,353	450	AUG/UAG	12	H
nd5	15,579	17,471	1,893	630	AUG/UAA	11	H
trnA(ugc)	17,492	17,564	73	–	–	20	H
cox2	17,626	18,342	717	238	GUG/UAG	61	H
trnF(gaa)	18,333	18,405	73	–	–	-10	H
rns	18,407	19,663	1,257	–	–	1	H
trnG(ucc)	19,669	19,740	72	–	–	5	H
trnV(uac)	19,835	19,906	72	–	–	94	H

STable 9. Composition and skewness in the mitochondrial genome of *Latrunculia biformis*.

Gene/Region	G	A	T	C	GC%	AT-skew	GC-skew	Length (bp)
atp6	19.7	25.9	43.3	11.2	30.9	-0.25197	0.27753	735
atp8	21.9	31.5	36.5	10.1	32.0	-0.07383	0.37143	219
atp9	28.3	23.6	36.3	11.8	40.1	-0.21127	0.41053	237
cox1	23.7	25.0	37.6	13.6	37.3	-0.20082	0.27022	1,557
cox2	23.0	29.6	35.6	11.9	34.9	-0.09208	0.32000	717
cox3	24.8	24.2	38.3	12.7	37.5	-0.22515	0.32432	789
cytb	22.2	26.8	40.4	10.7	32.8	-0.20103	0.35092	1,155
nd1	23.3	25.4	40.1	11.2	34.5	-0.22449	0.34925	972
nd2	23.4	27.3	40.0	9.4	32.8	-0.18901	0.42664	1,353
nd3	21.7	27.5	44.4	6.4	28.1	-0.23552	0.54455	360
nd4	23.1	24.2	41.6	11.0	34.2	-0.26360	0.35484	1,452
nd4L	21.0	32.7	39.7	6.7	27.7	-0.09677	0.51807	300
nd5	20.5	28.0	41.2	10.4	30.9	-0.19113	0.32650	1,893
nd6	23.2	24.1	44.2	8.3	31.7	-0.29443	0.47429	552
rnl	23.4	35.8	28.7	12.2	35.6	0.11056	0.31144	2,484
rns	25.4	33.7	30.3	10.7	36.0	0.05224	0.40839	1,257
tRNAs[a]	26.7	26.8	31.7	14.8	41.5	-0.08350	0.28591	1,761
IGRs[b]	23.9	32.4	31.6	12.1	36.0	0.01366	0.32737	2,173
mtDNA[c]	23.4	28.7	36.4	11.5	34.9	-0.11781	0.33976	19,950

[a] Totaling 24 tRNA genes.

[b] Encompassing 36 intergenic regions (Q_1 = 12,Q_2 = 22, Q_3 = 41, Q_4 = 499).

[c] Discounting 31 bp from overlapping genes.

STable 10. Features of the mitochondrial genome of *Neopodospongia tupecomareni*.

Gene	Position Start	Position End	Length (bp)	Amino acid	Start/Stop codon	Intergenic region (bp)	Strand
rnl	1	2572	2572	–	–	7	H
trnI(cau)	2584	2655	72	–	–	11	H
trnY(gua)	2671	2741	71	–	–	15	H
trnK(uuu)	2969	3041	73	–	–	227	H
atp8	3043	3270	228	75	AUG/UAA	1	H
atp6	3264	3998	735	244	AUG/UAA	-7	H
trnR(ucu)	4029	4100	72	–	–	30	H
trnR(ucg)	4219	4292	74	–	–	118	H
cox3	4404	5192	789	262	AUG/UAG	111	H
missing[a]	5194	5207	14	–	–	–	H
trnQ(uug)	5211	5285	75	–	–	18	H
trnW(uca)	5375	5445	71	–	–	89	H
trnL(uag)	5488	5560	73	–	–	42	H
trnN(guu)	5565	5635	71	–	–	4	H
cytb	5637	6791	1155	384	AUG/UAG	1	H
trnT(ugu)	6825	6897	73	–	–	33	H
atp9	6939	7175	237	78	AUG/UAA	41	H
trnS(gcu)	7189	7275	87	–	–	13	H
trnP(ugg)	7287	7359	73	–	–	11	H
nd4	7390	8838	1449	482	AUG/UAG	30	H
trnH(gug)	8850	8922	73	–	–	11	H
trnE(uuc)	8957	9028	72	–	–	34	H
nd6	9026	9601	576	191	GUG/UAG	-3	H
trnD(guc)	9809	9879	71	–	–	207	H
nd3	9912	10265	354	117	AUG/UAG	32	H
nd4L	10286	10585	300	99	AUG/UAA	20	H
trnY(aua)	10943	11013	71	–	–	357	H
cox1	11018	12577	1560	519	AUG/UAG	4	H
missing[a]	11302	11634	333	110	–	–	H
trnS(uga)	12568	12646	79	–	–	-10	H
trnC(gca)	12757	12828	72	–	–	110	H
nd1	13073	14044	972	323	AUG/UAG	244	H
trnL(uaa)	14049	14122	74	–	–	4	H
trnI(gau)	14128	14200	73	–	–	5	H
trnM(cau)	14229	14299	71	–	–	28	H
nd2	14312	15670	1359	452	AUG/UAG	12	H
nd5	15681	17573	1893	630	GUG/UAA	10	H
missing[a]	16515	16619	105	34	–	–	H
trnA(ugc)	17588	17660	73	–	–	14	H
cox2	17759	18475	717	238	GUG/UAG	98	H
trnF(gaa)	18466	18538	73	–	–	-10	H
rns	18539	19773	1235	–	–	0	H
trnG(ucc)	19774	19845	72	–	–	0	H
trnV(uac)	19904	19976	73	–	–	58	H

[a] Missing data (N) according to regions from the *Negombata magnifica* mitogenome (NC_010171).

STable 11. Composition and skewness in the mitochondrial genome of *Neopodospongia tupecomareni*.

Gene/Region	G	A	T	C	GC%	AT-skew	GC-skew	Length (bp)
atp6	19.9	27.1	41.5	11.6	31.4	-0.21032	0.26407	735
atp8	18.9	34.7	37.3	9.2	28.1	-0.03659	0.34375	228
atp9	25.3	25.7	38.8	10.1	35.5	-0.20261	0.42857	237
cox1[a]	18.2	20.2	31.2	9.1	27.3	-0.21348	0.33333	1,560
cox2	23.7	29.2	36.1	11.0	34.7	-0.10684	0.36546	717
cox3	24.0	24.6	40.1	11.4	35.4	-0.23922	0.35484	789
cytb	20.9	27.6	41.3	10.2	31.1	-0.19849	0.34262	1,155
nd1	23.8	25.8	40.6	9.8	33.5	-0.22291	0.41718	972
nd2	22.4	27.3	41.1	9.2	31.6	-0.20215	0.41725	1,359
nd3	20.1	26.8	44.9	8.2	28.3	-0.25197	0.42000	354
nd4	22.0	25.2	42.7	10.1	32.1	-0.25813	0.36774	1,449
nd4L	21.3	31.0	40.3	7.3	28.7	-0.13084	0.48837	300
nd5[a]	19.0	27.1	39.1	9.3	28.3	-0.18117	0.34206	1,893
nd6	22.4	27.3	44.3	6.1	28.5	-0.23786	0.57317	576
rnl	23.1	36.3	28.6	12.1	35.1	0.11803	0.31340	2,572
rns	25.1	33.7	29.7	11.5	36.6	0.06258	0.37168	1,235
tRNAs[b]	23.9	29.1	32.4	14.7	38.5	-0.05329	0.23796	1,832
IGRs[a,c]	22.5	34.6	36.3	5.9	28.4	-0.02476	0.58419	2,050
mtDNA	22.0	29.0	36.5	10.2	32.2	-0.11455	0.36901	19,983

[a] Missing data (N, %): *cox1* = 21.3; *nd5* = 5.5, IGRs = 0.7.
[b] Totaling 25 tRNA genes.
[c] Encompassing 35 intergenic regions (Q_1 = 11, Q_2 = 28, Q_3 = 74, Q_4 = 357).
[d] Discounting 30 bp from overlapping genes.
